# Yucasin DF, a potent and persistent inhibitor of auxin biosynthesis in plants

**DOI:** 10.1038/s41598-017-14332-w

**Published:** 2017-10-25

**Authors:** Shinichi Tsugafune, Kiyoshi Mashiguchi, Kosuke Fukui, Yumiko Takebayashi, Takeshi Nishimura, Tatsuya Sakai, Yukihisa Shimada, Hiroyuki Kasahara, Tomokazu Koshiba, Ken-ichiro Hayashi

**Affiliations:** 1 Department of Biochemistry, Okayama University of Science, Kitaku, Okayama city, Okayama, 700-0005 Japan; 2 Graduate School of Life Sciences, Tohoku University, Sendai city, Miyagi 980-8577 Japan; 3 RIKEN Center for Sustainable Resource Science, Yokohama city, Kanagawa 230-0045 Japan; 4 Graduate School of Bioagricultural Sciences, Nagoya University, Nagoya city, Aichi 464-8601 Japan; 5 Graduate School of Science and Technology, Niigata University, Nishiku, Niigata, 950-2181 Japan; 6 Kihara Institute for Biological Research, Yokohama City University, Yokohama, Kanagawa 244-0813 Japan; 7 Institute of Global Innovation Research, Tokyo University of Agriculture and Technology, Fuchu city, Tokyo, 183-8509 Japan; 8 Department of Biological Sciences, Tokyo Metropolitan University, Hachioji city, Tokyo, 192-0397 Japan

## Abstract

The plant hormone auxin plays a crucial role in plant growth and development. Indole-3-acetic acid (IAA), a natural auxin, is mainly biosynthesized by two sequential enzyme reactions catalyzed by TAA1 and YUCCA (YUC). TAA1 is involved in the conversion of tryptophan to IPA, and YUC catalyzes the conversion of IPA to IAA. We previously demonstrated that yucasin inhibits AtYUC1 enzyme activity and suppress high-auxin phenotype of YUC overexpression plants, although yucasin displayed weak effects on the auxin-related phenotype of wild-type plants. To develop more potent YUC inhibitors, various derivatives of yucasin were synthesized, and their structure–activity relationships were investigated. Yucasin difluorinated analog (YDF) (5-[2,6-difluorophenyl]-2,4-dihydro-[1,2,4]-triazole-3-thione) was identified to be a more potent YUC inhibitor than the original yucasin. YDF caused an auxin-deficient phenotype in Arabidopsis wild-type plants that was restored with auxin application. YDF was found to be highly stable regarding metabolic conversion *in vivo*, accounting for the potent activity of the inhibition of IAA biosynthesis in planta. Photoaffinity labeling experiments demonstrated that yucasin-type inhibitors bind to the active site of AtYUC1. YDF is a promising auxin biosynthesis inhibitor and is a useful chemical tool for plant biology and agrochemical studies.

## Introduction

The plant hormone auxin is a master regulator for plant growth and development. Indole-3-acetic acid (IAA), the predominant naturally occurring auxin, regulates diverse physiological processes in almost every aspect of plant growth and development, including embryo development, vascular differentiation, apical dominance and tropic responses to light and gravity^1–4^. The hormonal action of auxin can be spatiotemporally regulated by cellular auxin distribution that is modulated by the biosynthesis, directional transport and inactivation of auxin^3,5,6^. Auxin biosynthesis plays a crucial role in the initial step of the establishment of cellular auxin distribution in plants^2,5^.

**Figure 1:**
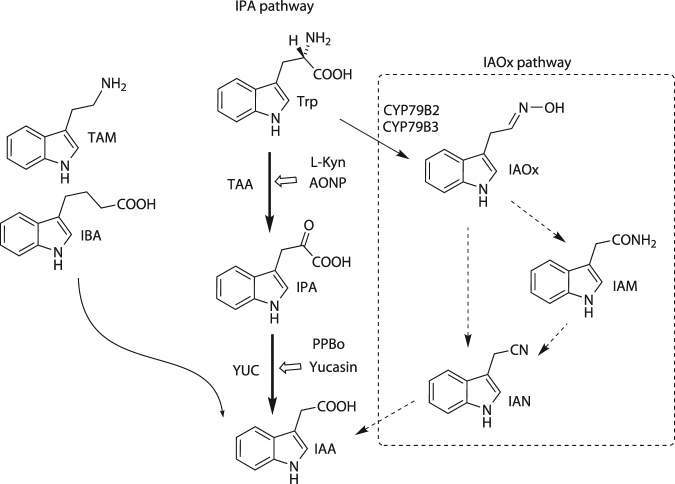
Auxin biosynthetic pathways and the structures of metabolic intermediates. indole-3-butyric acid (IBA), tryptamine (TAM), indole-3-acetaldoxime (IAOx), indole-3-acetonitrile (IAN), and indole-3-acetoamide (IAM).

IAA is mainly biosynthesized from tryptophan by two sequential enzymatic reactions consisting of *TRYPTOPHAN AMINOTRANSFERASE of ARABIDOPSIS 1* (*TAA1*) and *YUCCA* (*YUC*) of the indole-3-pyruvic acid (IPA) pathway (Fig. 1). TAA1 converts Trp to IPA, and YUC catalyzes the oxidative decarboxylation of IPA to produce IAA in the IPA pathway^7,8^. TAA1 and YUC orthologs are widely found in diverse land plants, such as the liverworts, mosses, ferns and vascular plants, and the IPA pathway is believed to be main IAA biosynthetic route in land plants^2,9,10^. YUC enzymes encode flavin monooxygenase and a part of a large gene family consisting of 11 members in *Arabidopsis* ^11,12^. Genetic studies have demonstrated that YUC functions as the rate-limiting enzyme of the IPA pathway, indicating the *YUC*s play a crucial role in developmental processes regulated by cellular IAA levels^5,13^. Molecular mechanisms underlying the regulation of cellular IAA levels still remain unclear due to the multiple regulatory steps involving the biosynthesis, directional transport and inactivation of auxin. In addition, the redundant functions of the large family of *YUC* genes prevent the access of reverse genetic approaches to understand the physiological role of local IAA biosynthesis. IAA-deficient mutants such as the *taa1 tar2* double mutant show severe impaired phenotypes^8^; therefore, it would be difficult to spatiotemporally analyze the role of IAA biosynthesis at a specific developmental stage.

Chemical biology approaches using small molecules have been effective at complementing genetic and biochemical approaches^14,15^. The function of target proteins can be spatiotemporally modulated by small molecules in any tissue or cell. Furthermore, small molecules can overcome the redundant activity of cognate genes and therefore can be an effective tool for studying diverse plant species inaccessible via genetic approaches. Recently, several auxin biosynthesis inhibitors targeting the IPA pathway have been discovered^16–18^. L-α-aminooxyphenylpropionic acid (AOPP) and its potent derivative pyruvamine (PVM) were reported to be TAA inhibitors^18^. TAA is pyridoxal phosphate (PLP)-dependent enzyme whose aminooxy moiety can bind PLP to inhibit oxime formation of PLP with substrate. PVM inhibits IAA biosynthesis and results in a severe auxin-deficient phenotype in several plants. L-Kynurenine (L-Kyn) has been reported to be a competitive inhibitor of TAA1 by chemical library screening^18,19^. L-Kyn is a metabolite of L-tryptophan via tryptophan dioxygenase^19^. Kakei *et al*. demonstrated that the phenyl boronic acid derivatives 4-biphenylboronic acid (BBo) and 4-phenoxyphenylboronic acid (PPBo) displayed very potent inhibition of the YUC enzyme and then repressed IAA biosynthesis, conferring an auxin-deficient phenotype to *Arabidopsis* plants^20^. In a previous study, we demonstrated that yucasin (5-[4-chlorophenyl]-2,4-dihydro-[1,2,4]-triazole-3-thione) was identified as a YUC inhibitor from synthetic chemical library. Yucasin effectively reduces the endogenous IAA level of maize and suppresses high-auxin phenotypes in *Arabidopsis* YUC overexpression plants. Yucasin also enhances the auxin-deficient phenotypes of *sav3–2/taa1* mutant plants. However, *Arabidopsis* wild-type plants treated with yucasin do not show typical auxin-deficient phenotypes^21^.

In this study, we synthesized various analogs of yucasin and investigated their structure–activity relationships (SARs). Among the various analogs, we identified a yucasin difluorinated analog (YDF: **1**) (5-[2,6-difluorophenyl]-2,4-dihydro-[1,2,4]-triazole-3-thione) as a potent, reversible YUC inhibitor using YUC-overexpressing transgenic plants harboring an auxin-responsive *DR5::GUS* reporter system. YDF caused an auxin-deficient phenotype in *Arabidopsis* plants and in lower land plants such as mosses and liverworts. The auxin-deficient phenotype caused by YDF was restored with auxin application. Based on the SAR results, we designed photoaffinity analogs of yucasin. Photoaffinity labeling experiments of AtYUC6 confirmed that yucasin-type inhibitors bind to the active site of YUC6 in competition with the substrate. Finally, we simultaneously blocked the IPA and CYP79B auxin biosynthesis pathways using a combination of the inhibitors and mutants for auxin biosynthesis and signaling, and the results suggested auxin null phenotype of *Arabidopsis* plants.

## Results

### Evaluation of inhibitory activity for yucasin analogs using AtYUC6 overexpression plants

**Figure 2:**
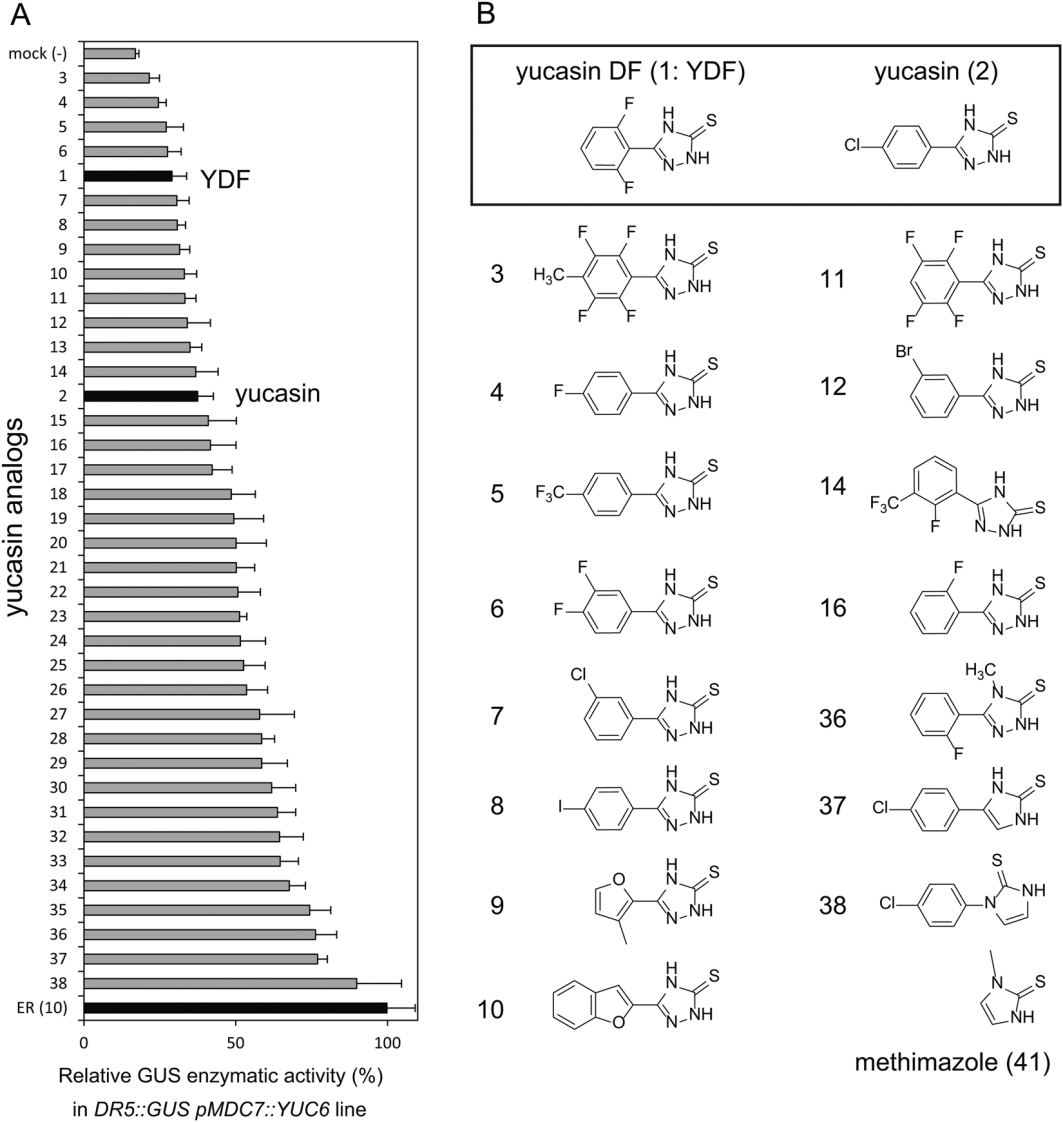
Structures of yucasin analogs and their inhibitory activity on *DR5::GUS* expression induced by *YUC6* overexpression. (**A**) 4-d-old *DR5::GUS pMDC7::YUC6* seedlings were incubated in the presence of 5 µM ER and 10 µM inhibitors for 20 h. ER-induced *YUC6* overexpression resulted in the accumulation of endogenous IAA and then promoted *DR5::GUS* expression. The induced GUS enzyme activity was fluorometrically determined and indicated as the relative values (%). Values are the means ± S.D. of three independent experiments. (**B**) Structures of yucasin analogs. The structures of all compounds are indicated in Supplemental Fig. S1.

Yucasin was identified as an inhibitor of the YUC enzyme in the IPA pathway. Yucasin suppressed high-auxin phenotypes of *Arabidopsis* YUC overexpression plants, but showed weak inhibitory activity on root growth of wild-type seedling (Supplemental Fig. S5)^21^. To develop a more potent auxin biosynthesis inhibitor, we synthesized various analogs of yucasin. In our previous study, the 1,2,4-triazole-3(4 *H*)-thione moiety of yucasin was found to be essential for the inhibition of the YUC enzyme^21^. On the other hand, the modification of the phenyl group may increase the ability of yucasin derivatives to bind to YUC enzymes. Therefore, we focused on the modification of the aromatic moiety of yucasin and synthesized analogs, as shown in Fig. 2 and Supplemental Fig. S1.

**Figure 3:**
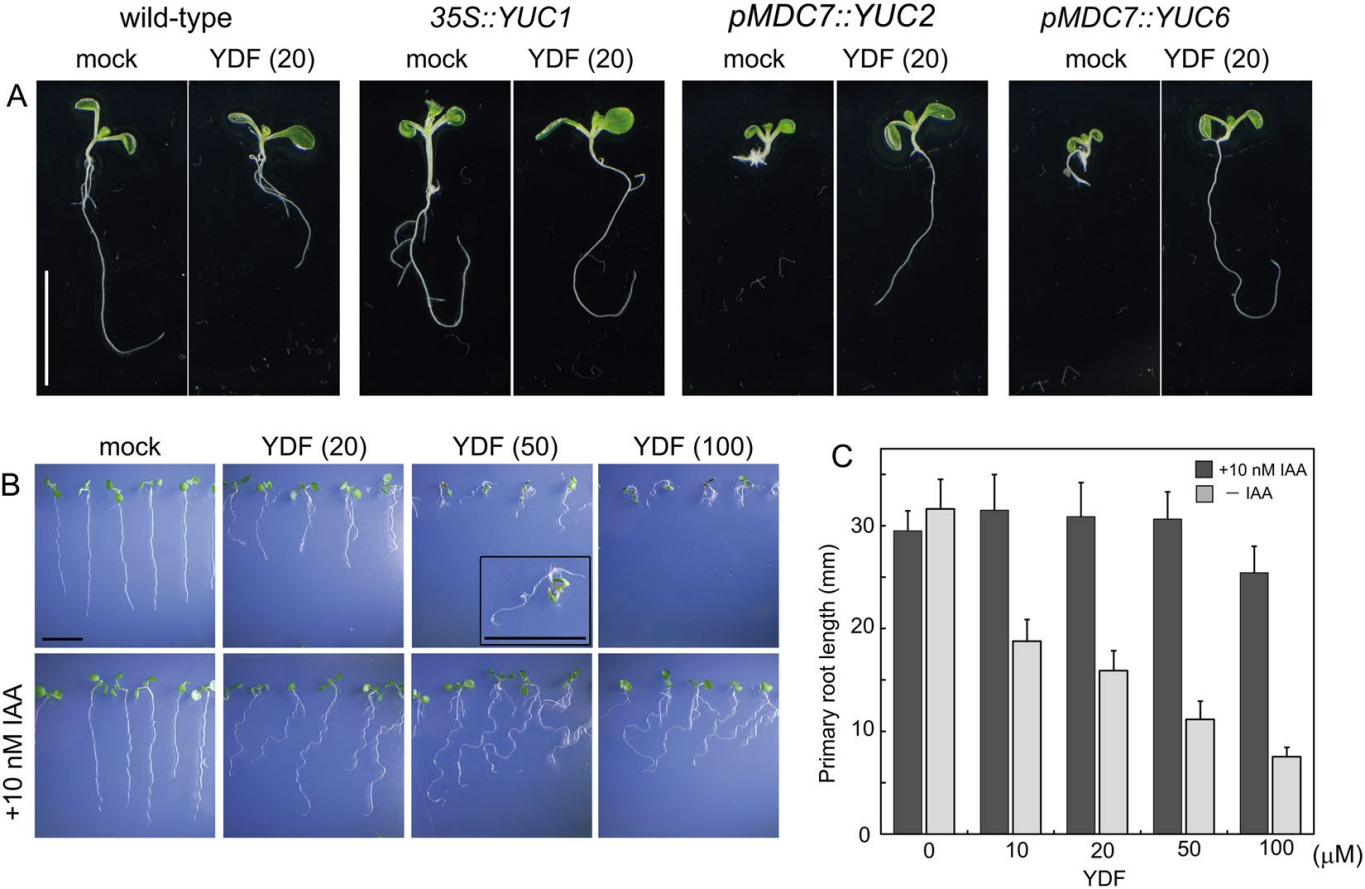
Effects of yucasin DF on wild-type and auxin overexpression plants. (**A**) Effects of YDF on high-auxin phenotypes resulting from *YUC1*, *YUC2* and *YUC6* overexpression. *35S::YUC1* plants were grown for 5 days in 1/2 MS medium in the presence of 20 µM YDF. *pMDC7::YUC2* and *pMDC7::YUC6* plants were cultured for 5 days in 1/2 MS medium in the presence of 5 µM estradiol (ER) and 20 µM YDF. The images were taken of representative phenotypes. Scale bar = 10 mm. (**B**) Effects of YDF on wild-type seedlings. The seedlings were grown vertically for 6 days on 1/2 MS agar plates with or without exogenous 10 nM IAA. Scale bar = 10 mm. (**C**) Inhibition of primary root growth by YDF in the presence or absence of 10 nM IAA. Root length was measured after 6 days of cultivation on vertical 1/2 MS agar plates. Values are the means ± S.D. (n = 30–35).

**Figure 4:**
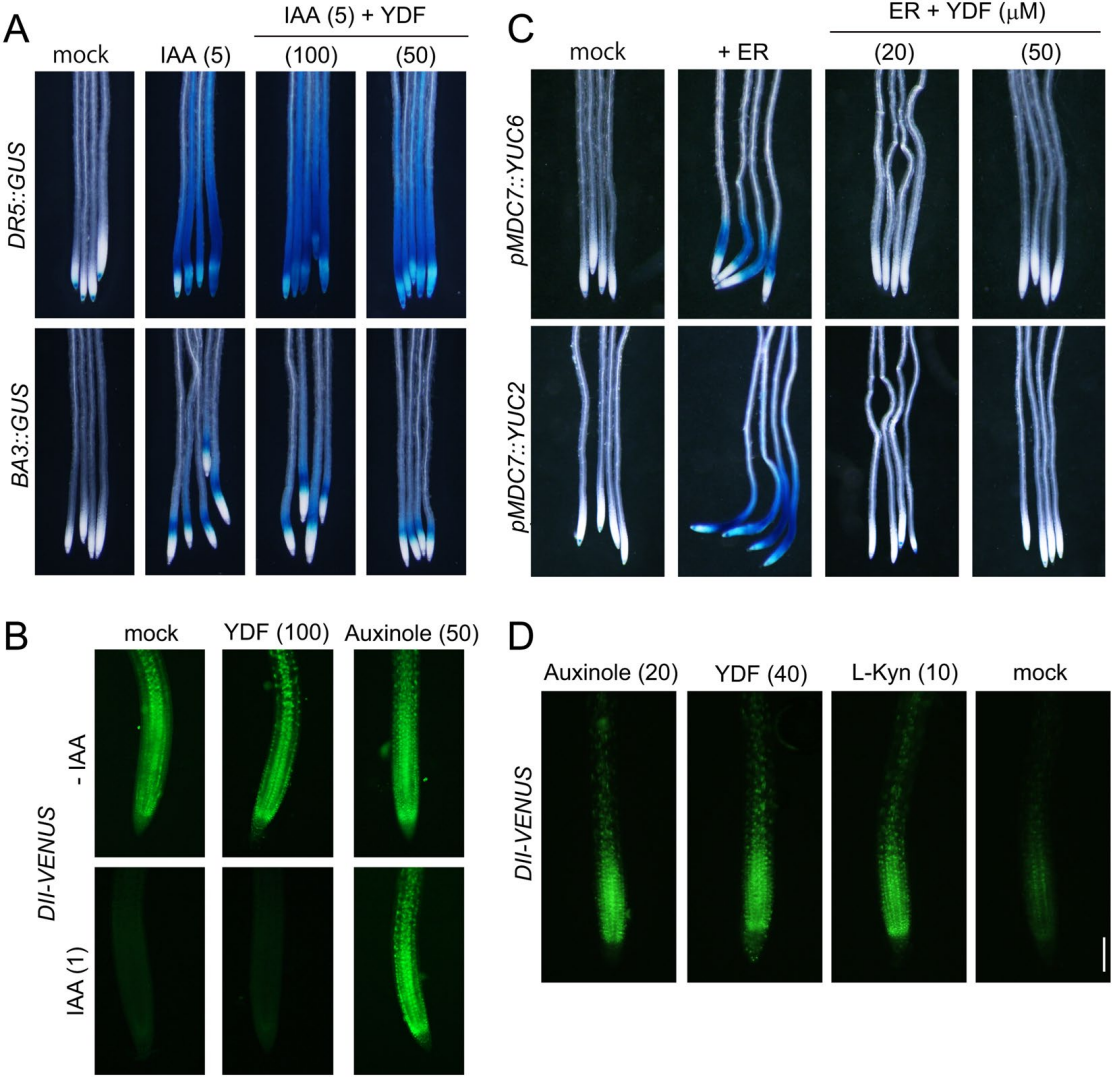
Effects of yucasin DF on the auxin-responsive gene expression and IAA biosynthesis. (**A** and **B**) Effects of YDF on the auxin-responsive gene expression regulated by the SCF^TIR1^ signaling pathway. (**A**) The auxin-responsive reporter lines *DR5::GUS* (upper) and *BA3::GUS* (bottom) expressed the GUS reporter gene in response to auxin. 5-d-old seedlings were treated with 5 µM IAA and YDF for 6 h. The values in parentheses indicate concentration (µM). (**B**) 6-d-old *DII-VENUS* seedlings were incubated with in the presence of YDF or auxinole in 1/2 MS liquid medium for 20 min. IAA (1 μM) was then added to medium, which was then incubated for an additional 40 min. The fluorescent images of root tips were immediately obtained using fluorescence microscopy. Scale bar = 100 µm. (**C** and **D**) Effects of YDF on IAA biosynthesis by IPA pathway. (**C**) 4-d-old *DR5::GUS pMDC7::YUC6* and *DR5::GUS pMDC7::YUC2* seedlings were incubated in the presence of 5 µM ER and YDF for 20 h. ER-induced *YUC* overexpression resulted in the accumulation of endogenous IAA and then promoted *DR5::GUS* expression. (**D**) 5-d-old *DII-VENUS* seedlings were incubated in the presence of inhibitors for 6 h. The auxin-dependent degradation of the *DII-VENUS* fusion protein was monitored by fluorescence microscopy.

In the initial evaluations for yucasin analogs, we measured the auxin-responsive *DR5::GUS* reporter expression that is up-regulated by endogenous IAA in *pMDC7::YUC6* lines^22^. The auxin-responsive expression of the *DR5::GUS* reporter was rapidly and specifically regulated by IAA levels (Fig. 2A)^23^. *pMDC7::YUC6*, an estradiol (ER)-inducible transgene, overproduced YUC6 enzymes in response to ER and consequently displayed extreme high-auxin phenotypes, as shown in Fig. 3A. ER at 5 μM induced *YUC6* expression to accumulate endogenous IAA, resulting in the activation of *DR5::GUS* expression (Fig. 4C). In this assay system, *DR5::GUS* in *pMDC7::YUC6* lines was incubated for 20 h with the analogs and ER. The induced GUS reporter expression was fluorescently quantified, and the inhibitory activities of the analogs were ranked, as shown in Fig. 2A. Consistent with previous results, the modification of the 1,2,4-triazole-3(4 *H*)-thione moiety (**36**–**38**) negated the YUC6 inhibitory activity. However, a clear correlation was not shown between the structural feature and inhibitory activity (Fig. 2 and Supplemental Fig. S1.). In this assay, 13 analogs showed more potent inhibitory activity than did yucasin.

We then evaluated the effects of analogs on the phenotype of *pMDC7::YUC6* (Fig. 3A and Supplemental Fig. S2). Four-day-old seedlings grown in the presence of ER exhibited extreme high-auxin phenotypes, including growth retardation of primary roots and shoots (Supplemental Fig. S2). Twelve analogs more potent than yucasin in the activation of *DR5::GUS* (Fig. 2), except for **5**, suppressed high-auxin phenotype of *pMDC7::YUC6* (Supplemental Fig. S2). Previous studies have demonstrated that yucasin suppressed high-auxin phenotypes in YUC overexpression plants, but wild-type plants treated with yucasin showed faint root growth inhibition (Supplemental Fig. S5)^21^. Therefore, the effects of analogs (**1**–**14**) on wild-type seedlings were examined. Analogs (**3**–**13** and YDF) affected the root growth of wild-type seedlings at 50 μM (Fig. 3B and Supplemental Figs S3 and S4). Auxin-deficient phenotypes could be rescued by exogenous auxin application if the defects were derived from the inhibition of IAA synthesis, not by off-target effects of compounds. Exogenous IAA (10–20 nM) was co-applied with analogs to evaluate the specificity of analogs on IAA biosynthesis. Fluorinated, chlorinated and furan analogs (**4**, **7**, **9**, **16**, YDF) showed reversible, potent inhibition of root growth in wild-type plants at 20 μM (Fig. 3B and Supplemental Fig. S3). L-Kyn was reported to be a competitive TAA inhibitor^19^. In our assay conditions, 50–100 μM L-Kyn showed an auxin-deficient like phenotype, but this impaired growth was partially recovered by exogenous IAA, implying off-target effects of L-Kyn at 50–100 μM (Supplemental Fig. S4). L-Kyn is the degradation product of L-Trp; therefore, L-Kyn might affect the metabolic pathway of Trp. Similar to L-Kyn, 6 analogs (**3**, **6**, **10**, **11**, **13** and **14**) displayed off-target effects, and the defects could not be rescued by IAA application (Supplemental Fig. S4).

From these selections of the candidates of potent YUC inhibitors, we found that yucasin 2,6-difluorinated (YDF) showed the most potent inhibitory activity *in planta* without off-target effects on root growth (Fig. 3B,C and Supplemental Fig. S5). In addition, YDF suppressed high-auxin phenotype, which displayed longer hypocotyls and epinastic cotyledons in *35S::YUC1* seedlings (Fig. 3A). The activity of the yucasin 2-monofluorinated analog (YMF: **16**) and furan analog (**9**) was similar but was slightly lower than that of YDF *in planta*. Therefore, we selected YDF as a promising candidate for the potent and specific inhibition of YUC and further investigated the mode of action of YDF.

### Yucasin DF inhibits auxin biosynthesis but not signal transduction processes

To confirm the specificity of YDF on auxin biosynthesis, we assessed the effects of YDF on auxin signaling by using two auxin-responsive reporter lines, *DR5::GUS* and *BA3::GUS* (Fig. 4A). The *DR5::GUS* and *BA3::GUS* transgene was rapidly induced by exogenous IAA via the auxin-dependent SKP1–CULLIN–F-box (SCF) complex-proteasome pathway. YDF at 100 μM showed no inhibition of *DR5::GUS* and *BA3::GUS* induction by exogenously applied IAA (Fig. 4A), but YDF completely repressed *DR5::GUS* expression by the overexpression of YUC2 and YUC6 enzymes (Fig. 4C). *DII-VENUS* plants constitutively express the DII-VENUS fusion protein (domain II of Aux/IAA repressor protein fused to the VENUS fluorescent reporter) under the control of the *35S* promoter (Fig. 4B and D)^24^. Auxin rapidly promotes the degradation of Aux/IAA repressors via SCF^TIR1^ auxin signaling pathway. Therefore, the *DII-VENUS* reporter has been widely used as an indirect marker to monitor cellular auxin levels. Exogenous 1 μM IAA triggered the rapid degradation of DII-VENUS during 40 min of incubation (Fig. 4B). Auxinole inhibited IAA-induced DII-VENUS degradation. In contrast, YDF did not inhibit the degradation of DII-VENUS induced by exogenous IAA (Fig. 4B). After 6 h of incubation with the chemicals, the TIR1 auxin receptor antagonist, auxinole caused the accumulation of DII-VENUS protein by inhibiting auxin signaling (Fig. 4D)^25^. L-Kyn and YDF also repressed the degradation of the DII-VENUS protein by blocking endogenous IAA synthesis (Fig. 4D). These results indicate that YDF is specific to the IAA biosynthesis pathway, but does not affect auxin-induced *DII-VENUS* degradation and auxin-responsive gene expression mediated by the SCF^TIR1^ auxin signaling pathway.

### Yucasin DF causes an auxin-deficient phenotype by inhibiting the IPA pathway

**Figure 5:**
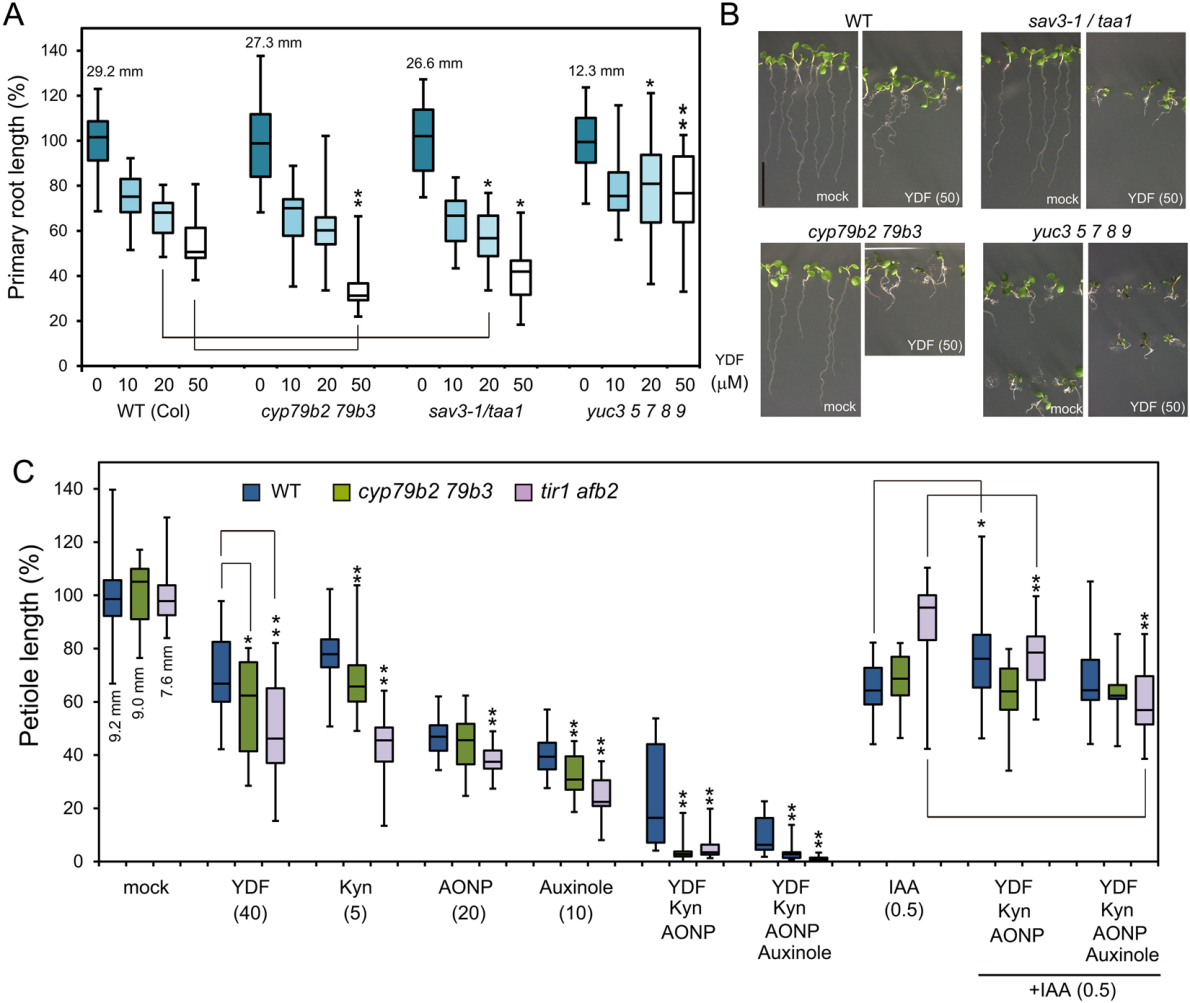
Effects of yucasin DF on the phenotypes of *Arabidopsis* auxin biosynthesis and signaling mutants. (**A**) Effects of YDF on root phenotypes of *Arabidopsis* auxin biosynthesis mutants. *Arabidopsis* seedlings were vertically grown for 6 days on 1/2 MS medium containing YDF. Primary root length of wild-type, *cyp79b2 79b3*, *sav3-1/taa1* and *yuc3 5 7 8 9* seedlings were measured. The relative root length was shown as the values (%) and a mock-treated root was adjusted to 100%. The actual length (mm) of mock-treated roots were indicated. Box-and-whisker plots show a median (centerline), upper/lower quartiles (box limits) and maximum/minimum (whiskers) n > 15. Statistical significance assessed by Welch’s two sample t-test. Asterisks indicate significant differences between WT and mutant at *P < 0.05 and **P < 0.01. (**B**) 6-d-old *Arabidopsis* wild-type, *cyp79b2 79b3*, *sav3-1/taa1* and *yuc3 5 7 8 9* seedlings grown vertically with 50 µM YDF. Scale bar = 10 mm. (**C**) Effects of YDF on shoot phenotypes of *Arabidopsis* auxin biosynthesis and signaling mutants. *Arabidopsis* wild-type, *cyp79b2 79b3* and *tir1 afb2* seedlings were cultured for 11 days on 1/2 MS plate containing TAA inhibitors [L-kynurenine (L-Kyn) and AONP], YUC inhibitor [YDF] and TIR1 antagonist [auxinole] with or without 0.5 μM IAA. The values in parentheses indicate concentration (µM). The petiole length of the first leaves was shown as relative value (100%) and the actual length (mm) of mock-treated plants. n > 18 Statistical significance assessed by Welch’s two sample t-test. *P < 0.05 and **P < 0.01.

To examine the effects of YDF on the growth of wild-type *Arabidopsis* plants, seedlings were grown vertically in the presence of YDF for 6 days. YDF showed inhibitory effects on primary root growth (Figs 3B,C, 5A and B), and 50 μM YDF phenocopied the auxin-deficient phenotypes of the auxin biosynthesis quintuple mutant *yuc 3 5 7 8 9* (*yucQ*) (Fig. 5A and B)^8,26^. This auxin-deficient phenotype of wild type caused by YDF was rescued by IAA application (Fig. 3 and Supplemental Fig. S6). The loss-of-functional *taa1* mutant, *sav3–1* was more sensitive to YDF than WT, suggesting that YDF targets the IPA pathway in a similar manner as yucasin (Fig. 5A). *Arabidopsis* has 11 functional *YUC* genes. The *yuc 3 5 7 8 9* quintuple loss-of-function mutant *yucQ* showed a very severe auxin-deficient phenotype in roots, as these YUC genes specifically function in the roots (Fig. 5A)^26^. The root phenotype of the *yucQ* mutant was further repressed by 10 μM YDF treatment (Fig. 5A). However, the root growth of the *yucQ* mutant was more resistant to 20–50 μM YDF in comparison with wild-type, implying the IPA pathway in *yucQ* mutant root was completely inhibited by 10 μM YDF.

The primary root growth of wild-type seedlings by YDF was fully restored by exogenous 5–10 nM IAA application (Fig. 3B,C and Supplemental Fig. S6), but wavy and slight agravitropic root phenotypes were observed. These root phenotypes were almost recovered by 20 nM IAA and 5 μM indole-3-acetamide (IAM), precursor of IAA (Supplemental Fig. S6). These results imply that optimal auxin concentration was different between root elongation and gravitropism.

We next investigated the effects of biosynthesis inhibitors including YDF on auxin biosynthesis mutants. IAA is mainly biosynthesized by two enzymes (TAA1 and YUC) in a linear IPA pathway. In addition, *Arabidopsis* has an auxiliary IAA biosynthetic pathway designated as the indole-3-acetaldoxime (IAOx) pathway, which is catalyzed by cytochrome P450 monooxygenase *CYP79B* ^5^. The chemical inhibition of biological processes, such as signaling and metabolic pathways, are sometimes accompanied by off-target effects, causing misleading experimental results. Off-target effects should especially be carefully considered when the pathway is completely blocked with high concentrations of inhibitors.

To achieve complete inhibition of the IPA pathway without off-target effects, we used a cocktail treatment for the inhibition of the IPA pathway. A cocktail treatment consisting of TAA1 inhibitors L-Kyn, AONP, and the YUC inhibitor YDF would be expected to completely block the IPA pathway (Fig. 5C and Supplemental Fig. S7). Wild-type plants treated with this inhibitor cocktail showed pleotropic defects; however, exogenous auxin application restored these defects (Fig. 5C and Supplemental Fig. S7), suggesting that these defects are not due to off-target effects of inhibitors. The loss-of-functional *cyp79b2 79b3* double mutant displayed more severe defects in the presence of YDF, L-Kyn and the biosynthesis inhibitor cocktail (Fig. 5A,C and Supplemental Fig. S7), suggesting that the IAOx pathway might partially complement auxin shortage by the inhibition of the IPA pathway. The *tir1 afb2* double mutant lacks two major auxin receptors of the six *TIR1/AFB* auxin receptors in *Arabidopsis*. The *tir1 afb2* mutant was more sensitive to biosynthesis inhibitors and auxin receptor antagonist, auxinole than wild-type (Fig. 5C). To examine the complete inhibition of auxin action in plants, auxin biosynthesis mutants were co-treated with L-Kyn, YDF, AONP and auxinole (Fig. 5C and Supplemental Fig. S7). Co-application with auxin biosynthesis inhibitors and auxinole caused extreme growth defects in wild type (Fig. 5C and Supplemental Fig. S7). This extreme growth defects of wild-type and mutants were restored by IAA application, suggesting the defects are derived from auxin null phenotype, but not from the toxic effects of inhibitors.

### Yucasin DF is stable derivative of yucasin

**Figure 6:**
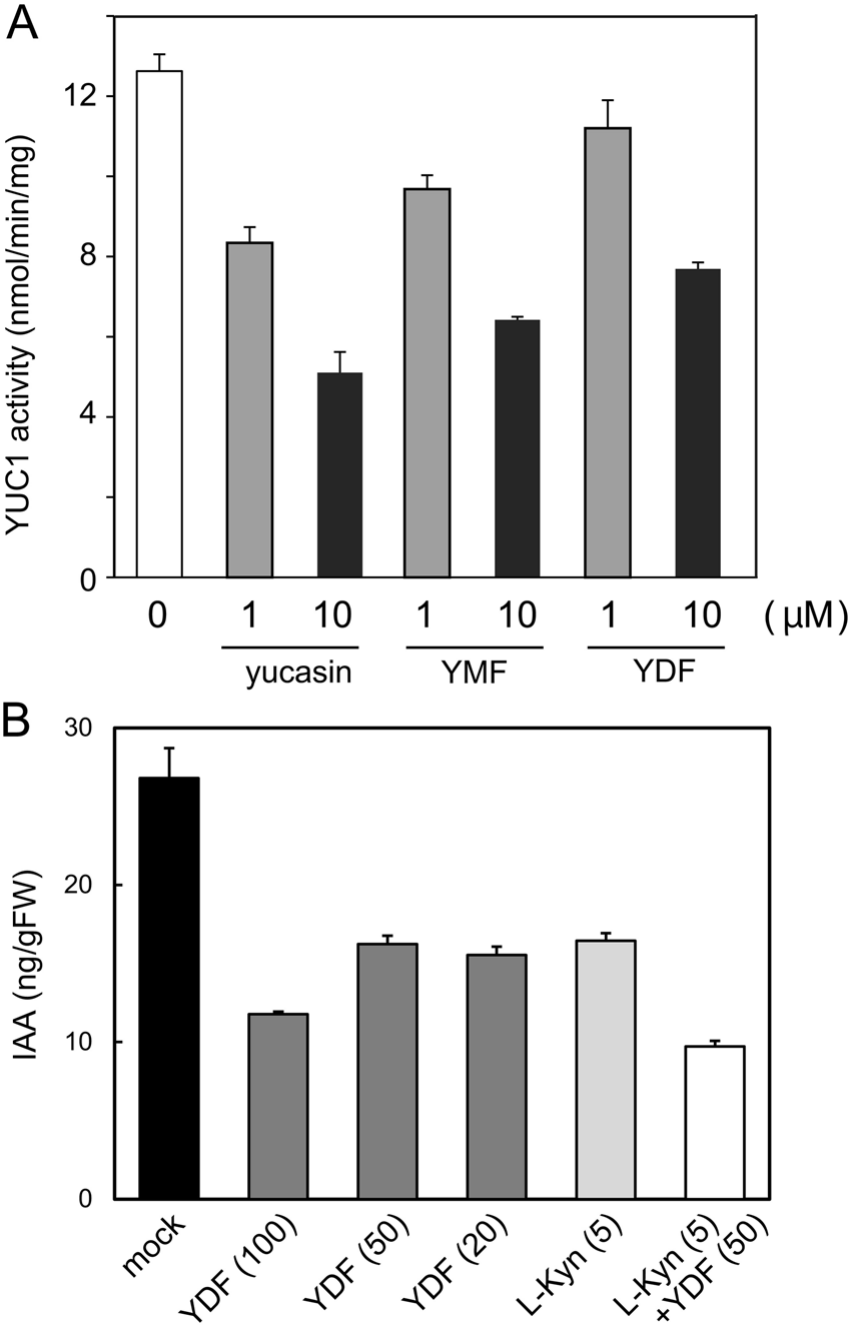
Effects of yucasin analogs on YUC1 recombinant enzyme activity and endogenous IAA levels. (**A**) Recombinant AtYUC1-His enzyme activity. The enzymatic reaction was performed using 0.25 µg of AtYUC1-His enzyme and 20 µM IPA at 35 °C for 30 min. The inhibitor was added at 1 or 10 µM. Values are the means ± S.D. of three independent experiments. (**B**) 5-d-old wild-type seedlings were incubated with inhibitor in liquid 1/2 MS medium for 10 h. The seedlings (n = 5–8) were pooled for each sample, and three samples were analyzed for each data point. Endogenous IAA levels were measured using LC-MS/MS. Values are the means ± S.D.

To unveil the mechanism of the potent activity of YDF in plants, we measured the inhibitory activity of YDF on the recombinant AtYUC1 enzyme (Fig. 6A). Surprisingly, YDF showed lower activity than yucasin. In addition, the yucasin 2-monofluorinated analog (YMF:16) showed more potent inhibition than YDF (Fig. 6A). In our previous study, 20 μM yucasin showed considerable reduction of endogenous IAA levels in *35S::YUC1* after 5 h of incubation; however, yucasin at 20 μM displayed a slight inhibition of endogenous IAA in wild-type plants^21^. In contrast to the weak effects of yucasin on endogenous IAA levels in wild-type plants, YDF at 20 μM showed potent inhibition in wild-type plants to similar extent to L-Kyn (5 μM) (Fig. 6B). Both 20 μM YDF and 5 μM L-Kyn showed auxin-deficient phenotypes (Fig. 5 and Supplemental Fig. S7), and co-treatment with L-Kyn and YDF showed the most potent inhibition of endogenous IAA levels (Fig. 6B).

Yucasin inhibited the recombinant YUC1 enzyme activity more potent than YDF, but showed very weak activity against the root growth of wild-type seedlings (Supplemental Fig. S5). Yucasin was demonstrated to suppress high-auxin phenotypes caused by the overexpression of *YUC1*, *YUC2*, and *YUC6* at same concentration range (20−50 μM) as that of YDF^21^. Thus, it is unlikely that yucasin would be a selective inhibitor of the YUC1 enzyme and not be active on other YUCs. The calculated log P values of yucasin and YDF were also similar (yucasin: 2.75 and YDF: 2.50), implying that the membrane permeability of these compounds are not different. Generally, the imine moiety is readily hydrolyzed to yield aryl hydrazide. An alternative explanation is that yucasin is a metabolically and chemically unstable compound. On the other hand, YDF is more stable *in vivo* and *in vitro* for maintaining a constant concentration during incubation.

**Figure 7:**
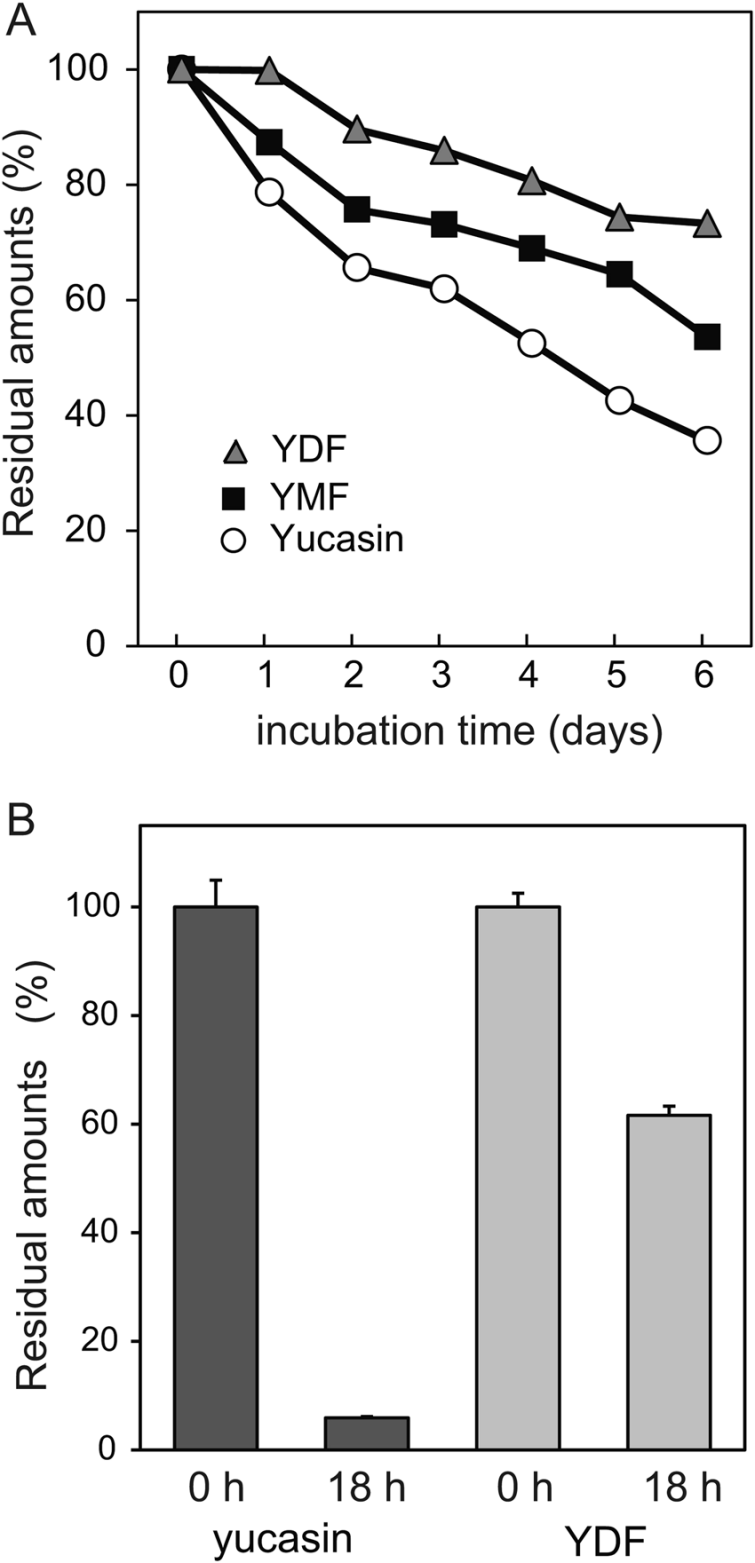
Chemical and metabolic stability of yucasin and fluorinated yucasin. (**A**) Chemical stability of the inhibitors in culture medium. The inhibitors (100 µM) were incubated in 1/2 MS liquid medium for 6 days at 24 °C under continuous light. The sample was analyzed using HPLC at regular intervals. The residual amount of inhibitor is shown as the relative value (%) compared with the initial amount. (**B**) Metabolic stability of the inhibitors in root cell lysate. The inhibitors (250 µM) in *Arabidopsis* root homogenate were incubated for 18 h at 24 °C in the dark. Residual inhibitors were measured using HPLC after the incubation. Values are the means ± S.D.

To confirm the chemical stability of the inhibitors, YDF, YMF, and yucasin were incubated in liquid culture medium, after which the residual amount was measured by HPLC at regular intervals during incubation. YDF was found to be most stable in the medium (Fig. 7A). Seventy-five percent of YDF and 50% of YMF still remained after 6 days of incubation. In contrast, less than 35% of yucasin remained after 6 days (Fig. 7A). These data suggest that the introduction of a fluorine group improves the chemical stability of yucasin-type inhibitors. To examine the metabolic stability of YDF and yucasin, the inhibitors were measured using HPLC after incubation for 18 h in *Arabidopsis* root cell lysate (Fig. 7B). Consistent with the chemical stability, YDF showed higher metabolic stability in the root cell homogenate. More than 90% of yucasin was metabolically converted; in contrast, 60% of YDF remained unchanged (Fig. 7B and Supplemental Fig. S8). This evidence indicates that YDF is a highly stable yucasin derivative and therefore shows potent inhibitory activity on auxin biosynthesis *in planta*.

### Yucasin DF inhibits the auxin biosynthesis of lower land plants such as the moss *Physcomitrella patens* and the liverwort *Marchantia polymorpha*

**Figure 8:**
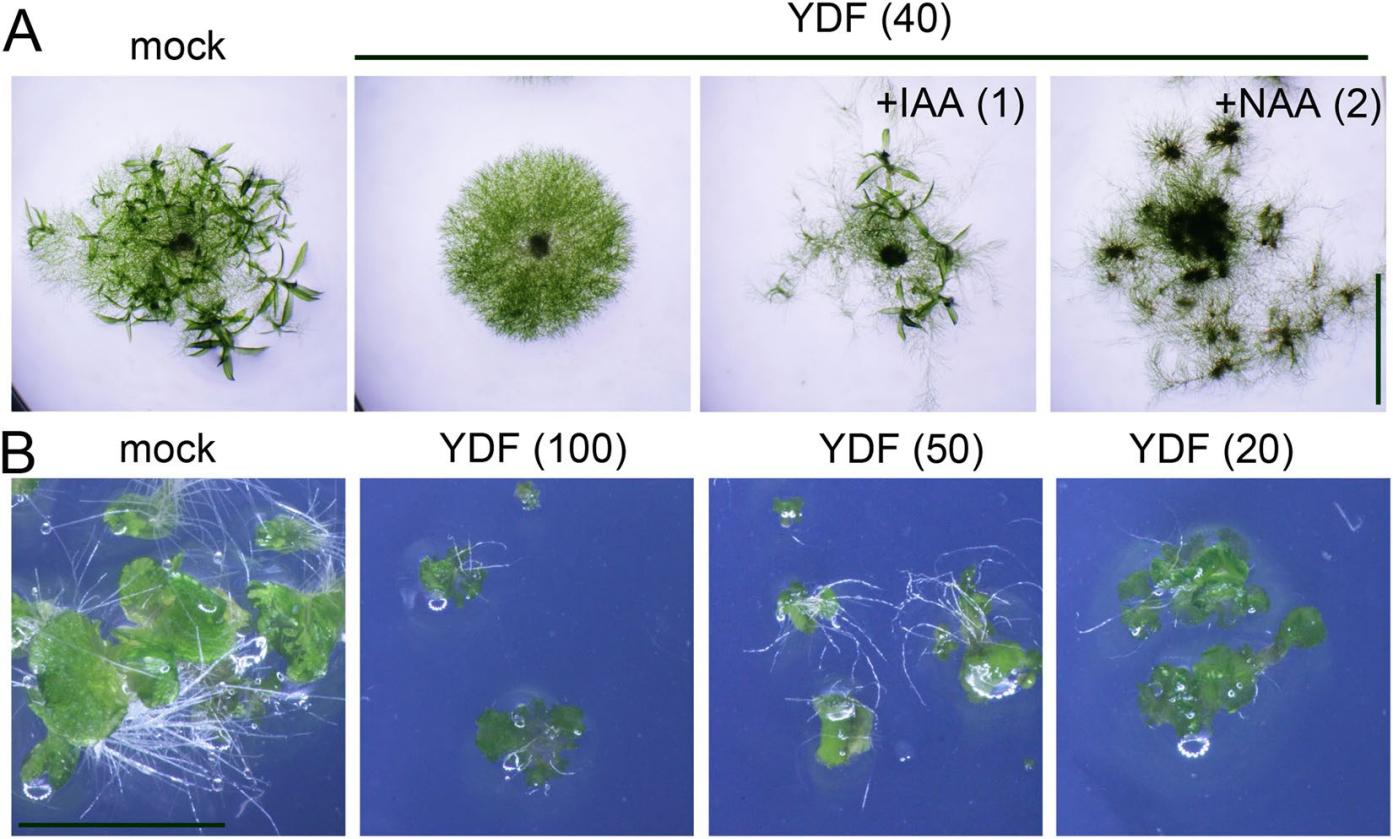
Effects of yucasin DF on the growth of the moss *Physcomitrella patens* and liverwort *Marchantia polymorpha*. (**A**) The protonemata of *P. patens* were placed on BCDAT plates containing 40 µM YDF with or without auxins (1 µM IAA and 2 µM NAA). The plates were cultured at 24 °C for 18 days under continuous light. Scale bar = 5 mm. (**B**) The gemmae of the liverwort *Marchantia polymorpha* were cultured on 1M51C plates with YDF for 11 days. Scale bar = 5 mm.

The IPA pathway is thought to be the predominant IAA biosynthetic pathway in land plants. The model moss plant *Physcomitrella patens* contains both *TAA* and *YUC* homologs responsible for the IPA pathway in the genome^10,27^. Auxin regulates the development of protonemata, which is the filamentous tissue of moss^9,28^. Exogenous IAA promotes the transition from chloronema to caulonema cells and then to the gametophyte. YDF inhibited protonema development (the transition to caulonema cells) and consequently repressed the formation of the gametophyte (Fig. 8A). The exogenous auxins IAA and NAA restored the auxin-deficient protonema phenotype and rescued the formation of the gametophyte (Fig. 8A). The liverwort *M. polymorpha* has a single *TAA* gene and two *YUC* genes. Eklund *et al*. reported that *taa* knockout lines showed very severe growth defects in the thallus. Consistent with the phenotype of the *taa* knockout line^10^, YDF strongly repressed the growth of the thallus in *M. polymorpha* (Fig. 8B). This evidence suggests that YDF can modulate IAA biosynthesis in diverse land plants.

### Yucasin directly binds to the active site of the YUC enzyme in competition with the IPA substrate

**Figure 9:**
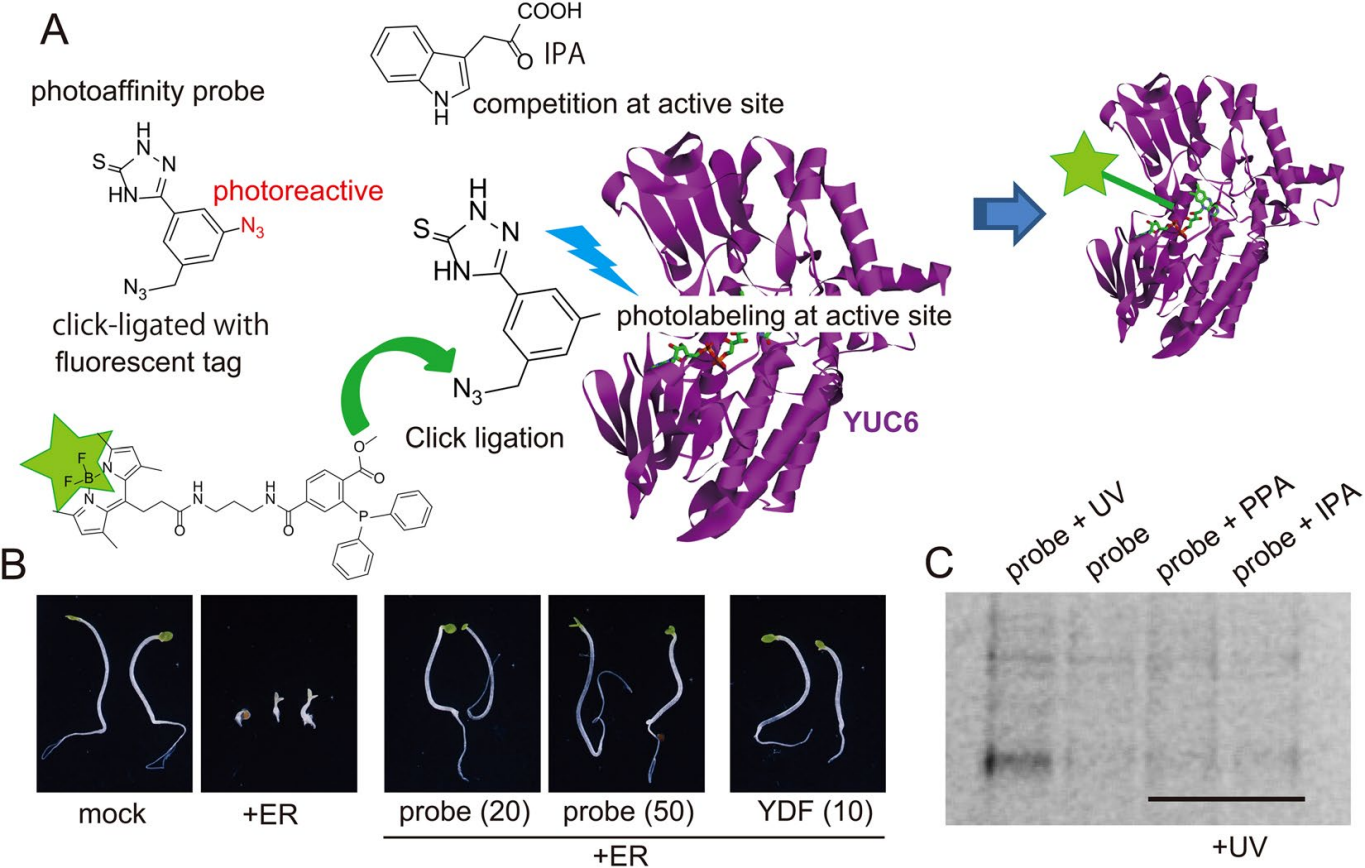
Photoaffinity labeling experiments of YUC6 by the bifunctional photoreactive yucasin probe. (**A**) Diagram of photoaffinity labeling experiment by the bifunctional photoreactive yucasin probe. The photoreactive aryl azido group (red) conjugates with YUC6 recombinant protein. After photoaffinity labeling, the alkyl azido group can ligate to the fluorescent phosphine tag in accordance with the Staudinger–Bertozzi reaction. YUC6 was fluorescently tagged by the conjugation with the probe. If the probe competes with the substrate phenylpyruvic acid (PPA) or indole-3-pyruvic acid (IPA) at active site, fluorescently tagged YUC6 cannot be detected. (**B**) Effects of the probe on high-auxin phenotype of *pMDC7::YUC6* etiolated seedlings. The etiolated seedlings were grown in 1/2 MS medium in the presence of inhibitors and 10 µM ER in the dark for 3 days. (**C**) Fluorescent gel image of SDS-PAGE under 488-nm excitation and 520-nm emission wavelengths. YUC6 was irradiated by UV in the presence of 20 µM probe (1^st^ lane from left), YUC6 was not irradiated in the presence of 20 µM probe (2^nd^ lane), YUC6 was irradiated by UV in the presence of 20 µM probe and 200 µM PPA (3^rd^ lane), and YUC6 was irradiated by UV in the presence of 20 µM probe and 200 µM IPA (4^th^ lane).

Yucasin was demonstrated to be a competitive inhibitor of the YUC1 enzyme in *in vitro* assays. To confirm the competitive binding of yucasin at the substrate binding site of YUC6, we performed photoaffinity labeling approach with the bifunctional photoaffinity yucasin probe (Fig. 9A). The structure-activity study for yucasin revealed that *m*-substituted analogs (**7**, **12**, and **15**) retain YUC inhibitory activity (Fig. 2 and Supplemental Fig. S1 and S2). The bifunctional photoaffinity probe has aryl azido and alkyl azido groups at the *m*-position of the phenyl ring of yucasin (Fig. 9A). The alkyl azido group can be photostable but specifically reacts with fluorescent groups tagged with triarylphosphine via Staudinger–Bertozzi ligation reaction^29^. The aryl azido group can be highly photoreactive and then conjugate with neighboring amino acid in the binding site of the target protein (Fig. 9A)^30,31^. The probe was synthesized and then evaluated using YUC6 and YUC1 overexpression lines. *pMDC7::YUC6* and *35S::YUC1* seedlings were grown under darkness with inhibitors. YUC6 and YUC1 overexpression caused the inhibition of hypocotyl elongation, a typical auxin response in etiolated seedlings (Fig. 9B and Supplemental Fig. S9). Both YDF and the probe inhibited high-auxin phenotype in etiolated seedlings and restored hypocotyl elongation (Fig. 9B), indicating that the probe could bind to the YUC6 enzyme. Recombinant YUC6 protein was incubated with the probe with or without the substrate (IPA and phenylpyruvic acid [PPA]) and photoactivated by UV irradiation. The YUC6 protein was precipitated with acidic acetone, after which the excess unreacted probe was removed. The fluorescent BODIPY group tagged with triarylphosphine was incubated with YUC6 protein to ligate with the alkyl azido group of the probe^32^. The YUC6 protein was analyzed by SDS-PAGE, and the probe-linked YUC6 protein was detected using a fluorescence laser imaging system. A fluorescent signal from the photoaffinity-labeled probe of YUC6 was detected (Fig. 9C, 1^st^ lane from left), but a signal from the probe linked with YUC6 was not detected without photoirradiation (Fig. 9C, 2^nd^ lane). The fluorescent signal disappeared when the probe was photoactivated in the presence of the substrates (Fig. 9C, 3^rd^ and 4^th^ lanes from left). This evidence indicates that yucasin-type inhibitors directly bind to the active site of the YUC enzyme in competition with the substrate.

## Conclusions

Based on the screening of various yucasin analogs, we identified yucasin DF as a specific and potent inhibitor of YUC. Yucasin-type inhibitors could be hydrolyzed *in planta* and are converted to thiosemicarbazide and corresponding carboxylic acid. Indeed, some aromatic carboxylic acids from analogs showed the inhibition of plant growth, suggesting that the stability of analogs may partly contribute to off-target effects on root growth. Methimazole (Fig. 2) has a similar structure of 1,2,4-triazole-3(4 *H*)-thione moiety in yucasin, and methimazole is reported to be an inhibitor of flavin-containing monooxygenases involving the YUC family^33,34^. We modified the 1,2,4-triazole-3(4 *H*)-thione moiety of yucasin analogs (**36**, **37** and **38**); however, all modifications resulted in the complete loss of inhibitory activity, indicating that the 1,2,4-triazole-3(4 *H*)-thione moiety is essential for binding to YUC. The structure–activity relationship (SAR) study of the phenyl moiety in yucasin analogs did not provide any rational regularity regarding the binding affinity of the analogs to the YUC enzyme. In our assay using the *DR5::GUS* reporter in *pMDC7::YUC6*, the analogs were evaluated as effective inhibitors *in planta*. Several factors such as the membrane permeability, metabolic stability and binding affinity of the analogs affected the inhibitory activity in the *in planta* assays. Our SAR results implied that the substitution of the phenyl ring of the analogs affects both the metabolic stability *in planta* and the binding affinity to YUC.

The naturally occurring auxin IAA is thought to be mainly biosynthesized by the IPA pathway. In addition, IAA is supplied from the intermediates IBA, IAM, TAM and IAN^2,5^. Phenylacetic acid (PAA) is recognized as an endogenous auxin, but the physiological function and biosynthetic pathway of PAA has not been unveiled^35^. In the Brassicaceae, IAA is also supplied from an alternative minor IAOx pathway involving CYP79B^36^. In *Arabidopsis* plants, 3 *TAA* genes and 11 *YUC* genes function in the IPA pathway, and 2 *CYP79B* genes are involved in the IAOx pathway. Other enzymes responsible for the minor pathway of IAA supplies have not been revealed. These complicated metabolic pathways and redundant gene families for IAA biosynthesis would prevent the access of genetic approaches to studying the IAA biosynthetic pathway of various plant species. We blocked the genetically assigned IAA synthetic pathways (the IPA and IAOx pathways) by the combination of TAA and YUC inhibitors and the *cyp79b2 79b3* double mutant. The inhibition of both IPA and IAOx pathways resulted in a more impaired phenotype than that from the inhibition of the IPA pathway alone, but the plants still slightly grew in the absence of both pathways. The auxin antagonist auxinole blocked the auxin response derived from the minor IAA supply and PAA. Co-treatment with YDF, L-Kyn and auxinole completely blocked plant growth after the cotyledons opened. This evidence implies that the minor IAA supply and another natural auxin such as PAA play a role in auxin-regulated plant development.

YDF was identified as a potent inhibitor *in planta*, but the *in vitro* enzymatic inhibitory activity of YDF was much lower than that of yucasin. We revealed that YDF is a metabolically stable analog of yucasin and therefore showed potent activity *in planta*. Photoaffinity labeling experiments confirmed that the yucasin-type inhibitor binds to the active site of the YUC enzyme. The substrate recognition residue of the binding site may be conserved among land plants, implying yucasin-type inhibitors may be effective on diverse kinds of land plants. YDF was demonstrated to be effective on mosses and liverworts in addition to *Arabidopsis* plants (Fig. 8). These results demonstrated that YDF is a promising chemical tool for auxin biology in diverse land plants.

## Materials and Methods

### Synthesis of the yucasin analogs and photoaffinity yucasin probe

Yucasin analogs were synthesized in accordance with method A or method B as described below. For method A, aryl carboxylic acid (2 mmol) and thiosemicarbazide (5 mmol) were dissolved in DMF (5 mL), after which 1-(3-dimethylaminopropyl)-3-ethylcarbodiimide hydrochloride (WSCD HCl, 2.4 mmol) was added. The solution was stirred at room temperature for 4 h. The reaction mixture was then poured into water (50 mL) and then acidified to pH 3–4 with 2 M HCl. The solution was kept at 4 °C for 1 h. The precipitate was then collected by filtration and washed with distilled water. The product was dried *in vacuo* to afford aryl hydrazinecarbothioamide. For method B, aryl acid chloride (2 mmol) was added dropwise to thiosemicarbazide (4 mmol) in THF (10 mL). The reaction mixture was then stirred at room temperature for 6 h. The resulting suspension was poured into water (50 mL) and then extracted with EtOAc twice. The combined EtOAc layer was thoroughly washed with water and then concentrated *in vacuo*. The resulting powder was suspended in water and collected by filtration. The powder was then dried *in vacuo* to afford aryl hydrazinecarbothioamide.

The aryl hydrazinecarbothioamide obtained using method A or B (0.4–1.5 mmol) was suspended in aqueous sodium hydroxide (3 equivalents of NaOH in 10 mL of water) and stirred at 90 °C for 1.5 h. The resulting mixture was added to water (50 mL) and then acidified with 2 M HCl. The precipitate was collected by filtration and washed with distilled water. The product was dried *in vacuo* to afford the corresponding yucasin analogs.

The synthetic procedure of the photoaffinity yucasin probe is presented in the supplemental methods. The yield, physicochemical and spectroscopic properties of all analogs and probes are also provided in the supplemental methods.

### Plant materials and growth conditions

*Arabidopsis thaliana* (Col-0) was used as the wild type. Seeds were surface-sterilized and grown on solid medium (half-strength MS medium containing 1.2% sucrose and 4 g/L agar for agar medium or 14 g/L agar for vertical agar plates, pH 5.7). Primary root length was measured using ImageJ software. The wild-type strain of *P. patens* was cultured on BCDAT agar medium under continuous white light at 24 °C. *M. polymorpha* was cultured on half-strength Gamborg’s B5 media containing 8 g/L agar under continuous white light at 24 °C.

### Histochemical and quantitative GUS measurements

For fluorescent quantitative measurements of GUS reporter activity, 5-d-old *DR5::GUS pMDC7::YUC6* lines (n = 12−15) were homogenized in an extraction buffer as previously described^37^. After centrifugation, GUS activity was fluorophotometrically determined with 1 mM 4-methyl umbelliferyl-β-D-glucuronide as a fluorogenic substrate at 37 °C. For GUS histochemical analysis, plants were transferred to a GUS staining buffer (100 mM sodium phosphate [pH 7.0], 10 mM EDTA, 0.5 mM K_4_Fe(CN)_6_, 0.5 mM K_3_Fe(CN)_6_, and 0.1% Triton X-100) containing 1 mM 5-bromo-4-chloro-3-indolyl-β-D-glucuronide (X-Gluc). The plants were incubated at 37 °C until sufficient staining developed.

### Recombinant YUC1 enzyme assays

Recombinant YUC1 in the pET-53-DEST vector (Novagen, Japan) was expressed in the *E. coli* BL21 Star (DE3) strain (Invitrogen), and then YUC1 was purified using TALON metal affinity resin (Clontech). The enzyme assay was performed in a 50-µL reaction mixture containing 0.25 µg of YUC1, 20 µM IPA, 40 µM FAD, and 1 mM NADPH in 10 mM Tris-HCl buffer (pH 7.5) with or without inhibitors. IPA and inhibitors were added to the mixture immediately before the reaction. An assay without NADPH was used as a blank control to estimate the nonenzymatic conversion of IPA to IAA. Enzyme reactions were stopped by the addition of 50 µL of acetonitrile with ^13^C_6_-IAA (150 pmol) (Cambridge Isotope Laboratories) and analyzed using LC-MS/MS on TripleTOF 5600 (AB SCIEX) and Nexera UHPLC (Shimadzu) systems equipped with an HSS T3 column (1.8 µm, 2.1 × 50 mm) (Waters). Elution was carried out using 0.05% (v/v) acetic acid/water (solvent A) and 0.05% (v/v) acetic acid/acetonitrile (solvent B) with a gradient from 0% to 35% of solvent B (0 min to 6 min) at a flow rate of 0.4 mL/min. The temperature of the column was 40 °C. MS/MS analysis was performed in negative ion mode with the following parameters: declustering potential, −50; collision energy, −15; GS1, 40; GS2, 40; temperature, 500; and parent ion (*m/z*), 174.1 for IAA and 180.1 for ^13^C_6_-IAA. Fragment ions (*m/z*) of 130.06 and 136.08 were used for the quantification of IAA and ^13^C_6_-IAA, respectively.

### Measurements of endogenous IAA

LC-ESI-MS/MS analysis of IAA was performed using an Agilent 6420 Triple Quad system (Agilent) as previously described^35^.

### Stability of yucasin analogs in medium and root cell lysate

For chemical stability, yucasin, yucasin MF and yucasin DF (100 μM) were incubated in 1/2 MS liquid medium for 6 days at 24 °C under continuous light. The sample was analyzed using HPLC at regular intervals. The analytical conditions and HPLC chromatogram of yucasin and YDF are indicated in Supplemental Fig. S8. For metabolic stability, *Arabidopsis* roots (50 mg) were homogenized in 1.5 mL of 100 mM phosphate buffer (pH 7.0). After centrifugation, yucasin and YDF (250 µM) were incubated for 18 h at 24 °C in *Arabidopsis* root lysate in the dark. Residual inhibitors were measured using HPLC as indicated in Supplemental Fig. S8.

### Photoaffinity labeling experiments

Photoaffinity probes (20 μM) and substrate (200 μM) were added to the purified recombinant AtYUC6 enzyme solution (15 μg of YUC6 protein in 100 µL of 10 mM phosphate buffer), which was then kept in the dark for 30 min on ice. A 6-W UV hand lamp (UVG-54, UVP Co., Ltd) was placed over the sample at a 2-cm distance from the tube. The sample on ice was irradiated with 254-nm UV for 10 min. Two microliters of 2-mercaptoethanol (100 mM) was immediately added after irradiation. Cold acidic acetone (1% of 6 M HCl in acetone) was added to the YUC6 solution, after which the solution was kept for 4 h at −30 °C. The sample was then centrifuged (13,000 × g for 15 min), and the precipitated YUC6 protein was washed again with cold acetone to remove unreacted photoaffinity probe. The YUC6 protein was dissolved in 50 mM Tris-HCl buffer (pH 7.2, 50 μL) and then heated at 90 °C for 1–2 min. Fluorescent-tagged triaryl phosphine (50 μM final concentration) was added to react with alkyl azido groups at 40 °C for 5 h. After reacting, the YUC6 protein was again precipitated with cold acidic acetone (1% of 6 M HCl in acetone) and then washed with cold acetone to remove unreacted fluorescent tag molecules. The resultant tagged YUC6 protein was analyzed using SDS-PAGE and visualized using an FM-BIO II fluorescence imaging system (488 nm excitation and 520 nm emission wavelengths, Hitachi, Japan).

## Electronic supplementary material

Supplemental Information
