# Supplementary material for: Yucasin DF, a potent and persistent inhibitor of auxin biosynthesis in plants
Source: Sci Rep. 2017 Oct 25;7:13992. doi: 10.1038/s41598-017-14332-w (PMC5656596; doi:10.1038/s41598-017-14332-w)
Supplement: Supplementary file 1 — Supplemental Information [file 41598_2017_14332_MOESM1_ESM.pdf]

Supplementary Information

Yucasin DF, a potent and persistent inhibitor of auxin biosynthesis in plants

Shinichi Tsugafune[1], Kiyoshi Mashiguchi[2], Kosuke Fukui[1], Yumiko Takebayashi[3], Takeshi Nishimura[4], Tatsuya Sakai[5], Yukihisa Shimada[6], Hiroyuki Kasahara[3,7], Tomokazu Koshiba[8], Ken-ichiro Hayashi[1]*

Affiliations

[1] Department of Biochemistry, Okayama University of Science, Kitaku, Okayama city, Okayama 700-0005, Japan

[2] Graduate School of Life Sciences, Tohoku University, Sendai city, Miyagi 980-8577, Japan

[3]RIKEN Center for Sustainable Resource Science, Yokohama city, Kanagawa 230-0045, Japan Institute of Global Innovation Research

[4]Graduate School of Bioagricultural Sciences, Nagoya University, Nagoya city, Aichi 464-8601, Japan

[5]Graduate School of Science and Technology, Niigata University, Nishiku, Niigata, 950-2181 Japan

[6]Kihara Institute for Biological Research, Yokohama City University, Yokohama, Kanagawa 244-0813, Japan

[7]Institute of Global Innovation Research, Tokyo University of Agriculture and Technology, Fuchu city, Tokyo 183-8509, Japan

[8]Department of Biological Sciences, Tokyo Metropolitan University, Hachioji city, Tokyo 192-0397, Japan

***Corresponding author**

Correspondence to hayashi@dbc.ous.ac.jp

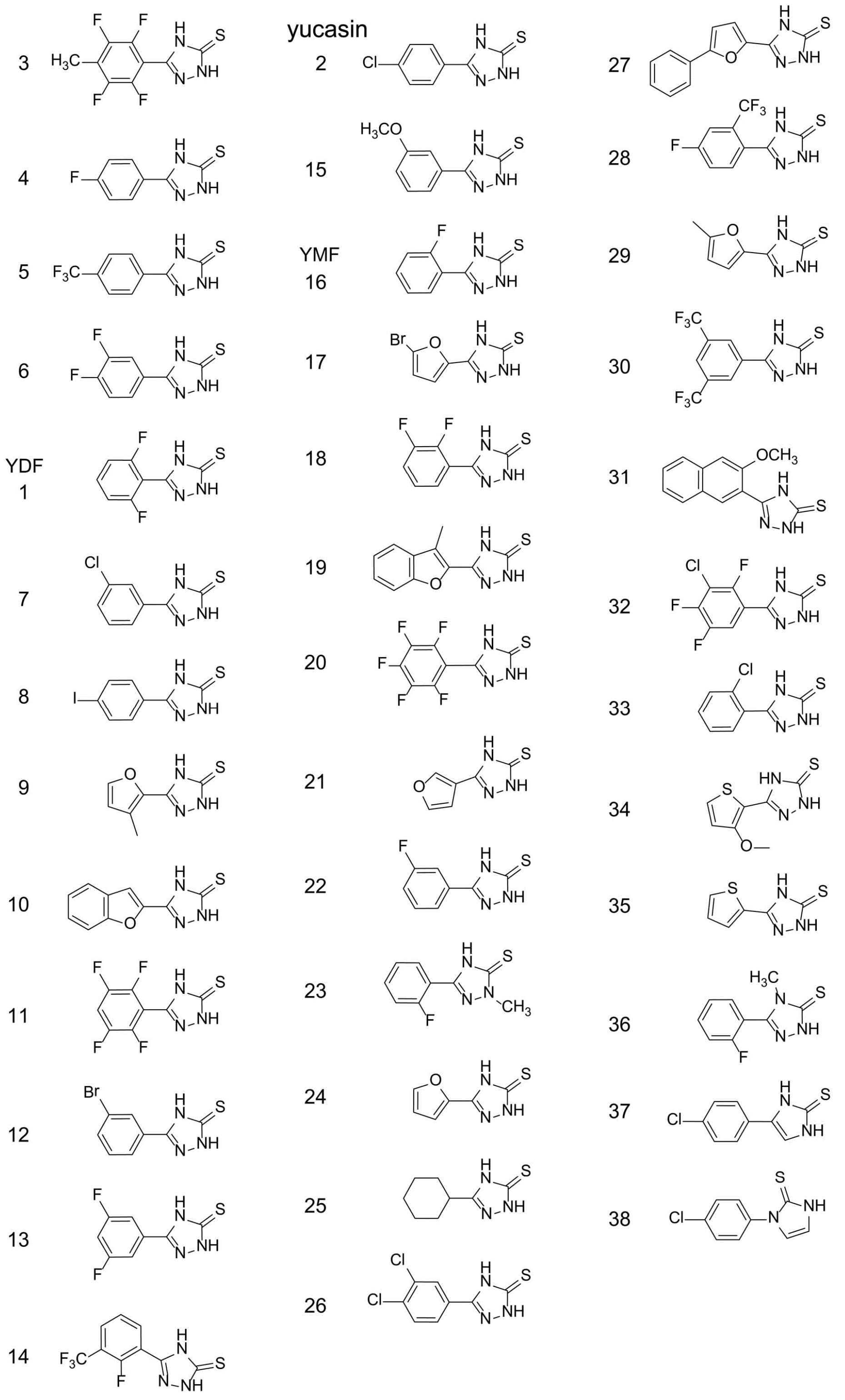

Supplemental Figure S1. Structures of yucasin analogs in this study.

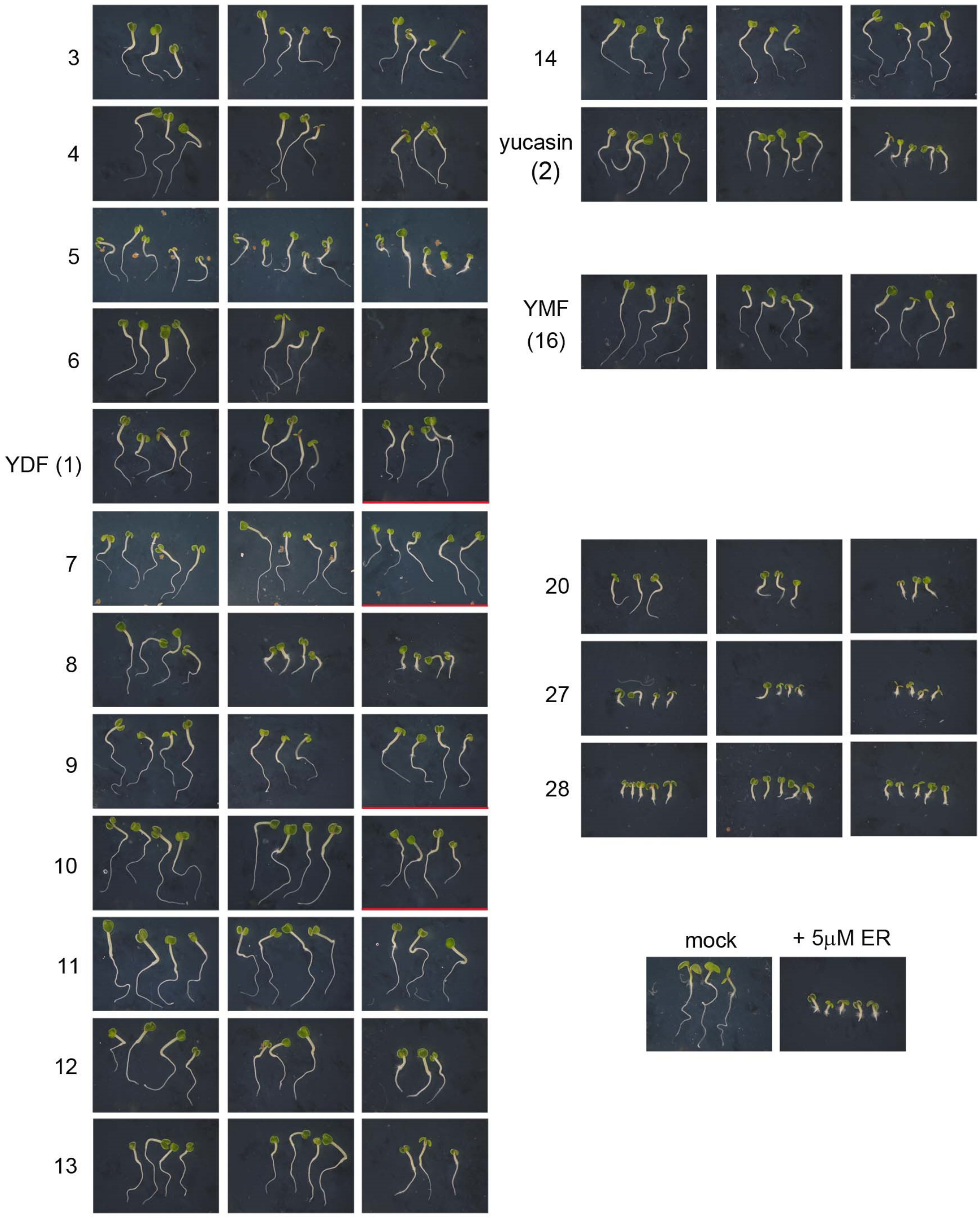

Supplemental Figure S2. Effects of yucasin analogs on the high-auxin phenotype in estradiol-inducible *YUC6* overexpression plants.

Arabidopsis pMDC7::YUC6 seedlings were grown for 4 days in 1/2 MS medium in the presence of 5 μM estradiol (ER) and yucasin analogs (20, 10, and 5 μM from the left panel). The induction of the YUC6 enzyme resulted in extreme high-auxin phenotypes. Yucasin and the active analogs (**1**−**38**) inhibited YUC6 activity to restore the high-auxin phenotype of the seedlings. The inactive analogs, such as **20**, **27** and **28**, failed to restore the high-auxin phenotype of *pMDC7::YUC6*.

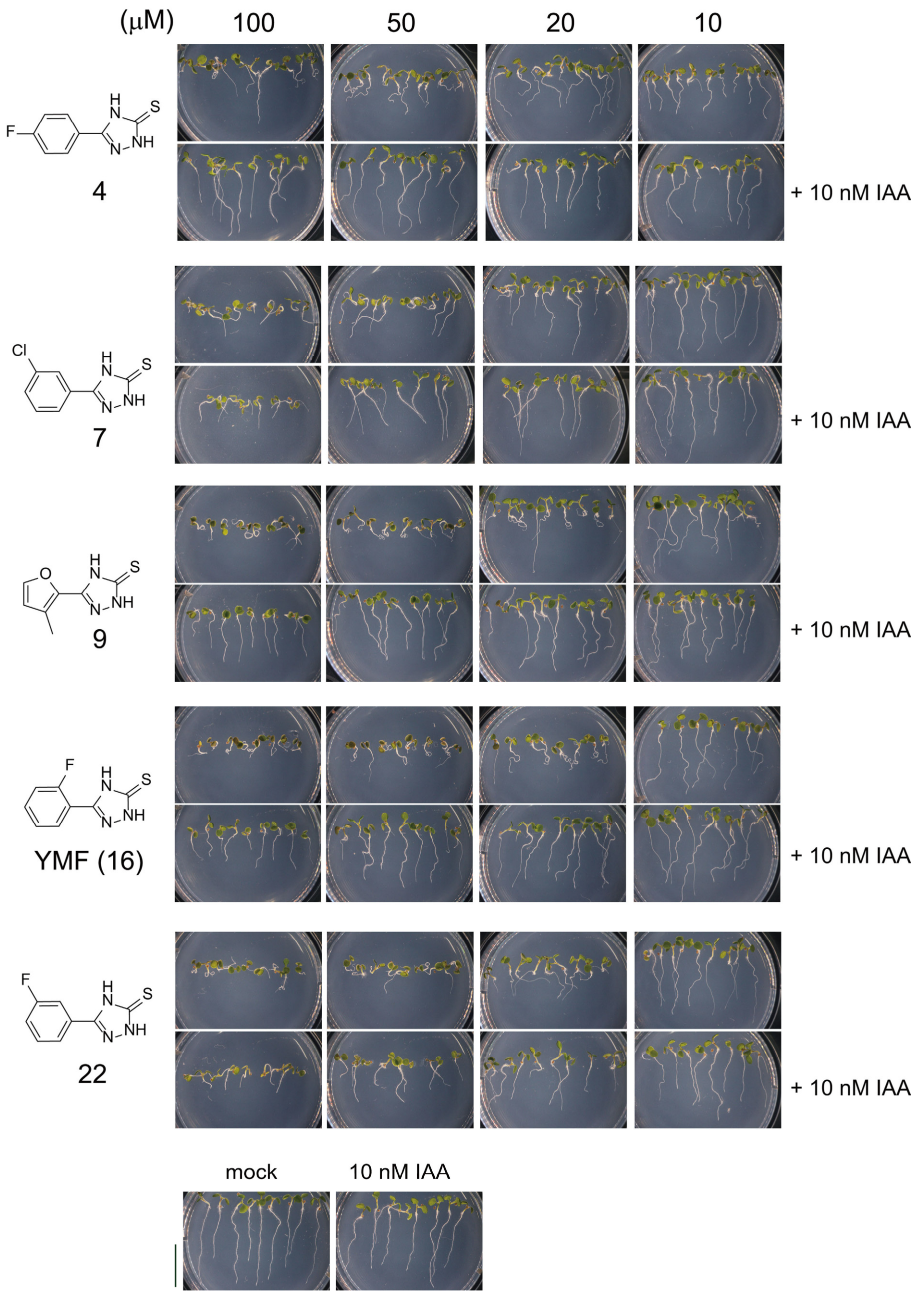

Supplemental Figure S3. Effects of yucasin analogs on wild-type seedlings.

Arabidopsis seedlings were grown vertically for 5 days in 1/2 MS agar plate containing the analogs in the presence or absence of 10 nM IAA. The impaired root phenotypes of analogs were rescued by exogenous IAA. Scale bar = 10 mm.

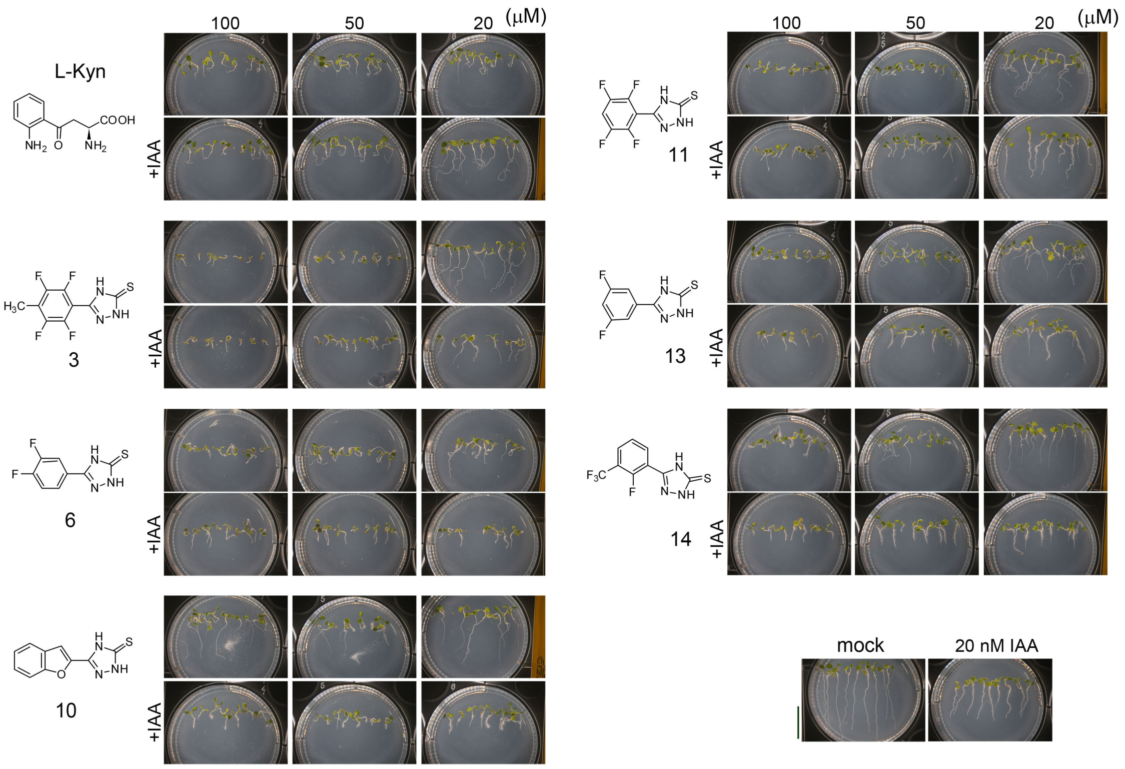

Supplemental Figure S4. Off-target effects of yucasin analogs and L-Kyn on wild-type seedlings.

Arabidopsis seedlings were grown vertically for 5 days in 1/2 MS agar medium containing compounds in the presence or absence of 20 nM IAA. The impaired root phenotypes caused by the compounds were not rescued by exogenous IAA. Scale bar = 10 mm

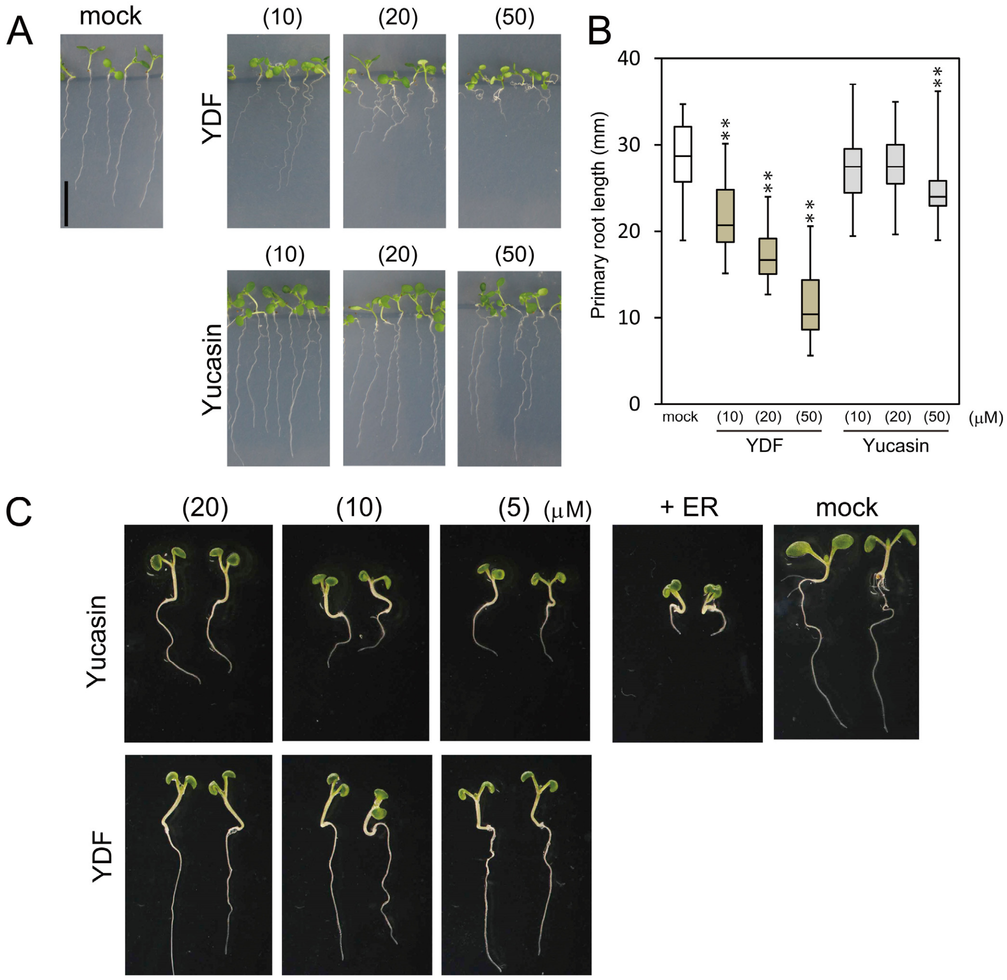

Supplemental Figure S5. Effects of yucasin and YDF on auxin-related phenotypes.

(A) *Arabidopsis* wild-type seedlings were grown vertically for 6 days on 1/2 MS agar plates with yucasin or YDF. Scale bar = 10 mm. (B) Primary root length of 6-d-old *Arabidopsis* wild-type seedlings grown vertically with yucasin or YDF. Box-and-whisker plots show a median (centerline), upper/lower quartiles (box limits) and maximum/minimum (whiskers). n>15. Statistical significance assessed by Welch's two sample t-test. Asterisks indicate significant differences between mock and the treatment with inhibitors at **P<0.01. (C) Effects of yucasin and YDF on the high-auxin phenotype of *YUC2* overexpression plants. *pMDC7::YUC2* seedlings were cultured for 5 days in 1/2 MS medium in the presence of 5 μM ER and inhibitors. The images taken are representative phenotypes. The values in parentheses indicate concentration (μM).

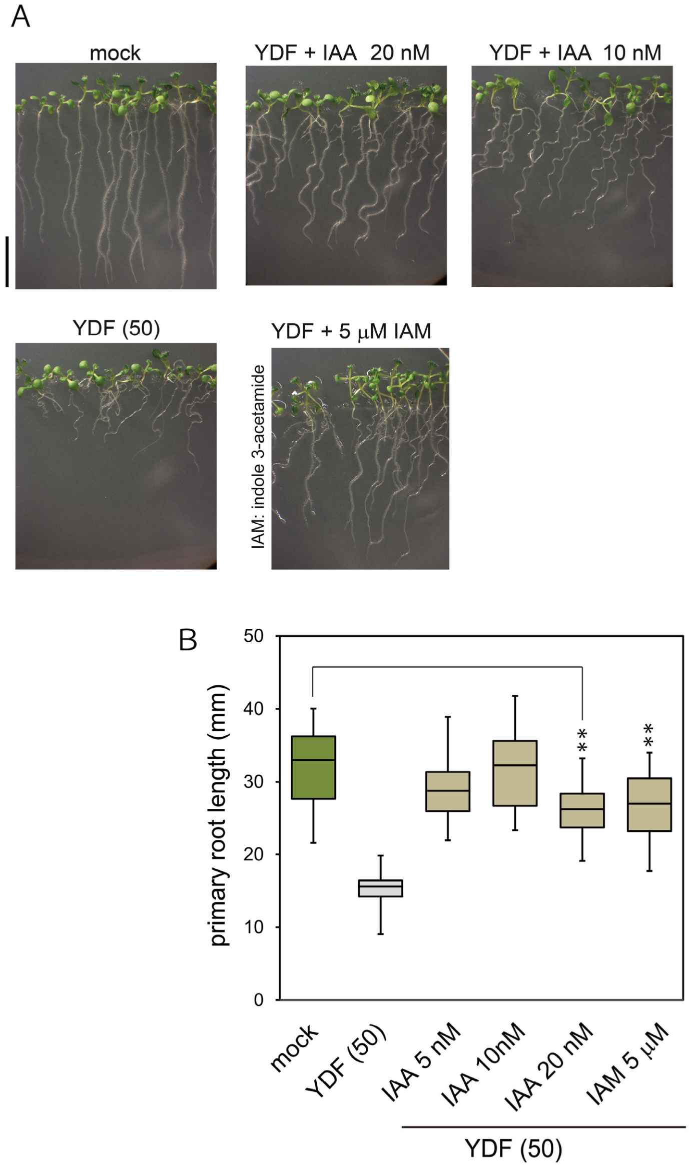

Supplemental Figure S6. Effects of exogenous auxin on auxin-deficient root phenotypes by YDF treatment.

(A) *Arabidopsis* wild-type seedlings were grown vertically for 6 days on 1/2 MS agar plates with yucasin and auxins. Scale bar = 10 mm. (B) Primary root length of 6-d-old *Arabidopsis* wild-type seedlings grown vertically with IAA and indole 3-acetamide (IAM) in the presence of 50 μM YDF. Box-and-whisker plots show a median (centerline), upper/lower quartiles (box limits) and maximum/minimum (whiskers). n>15. Statistical significance assessed by Welch's two sample t-test. Asterisks indicate significant differences between mock and the treatment with compounds at **P<0.01.

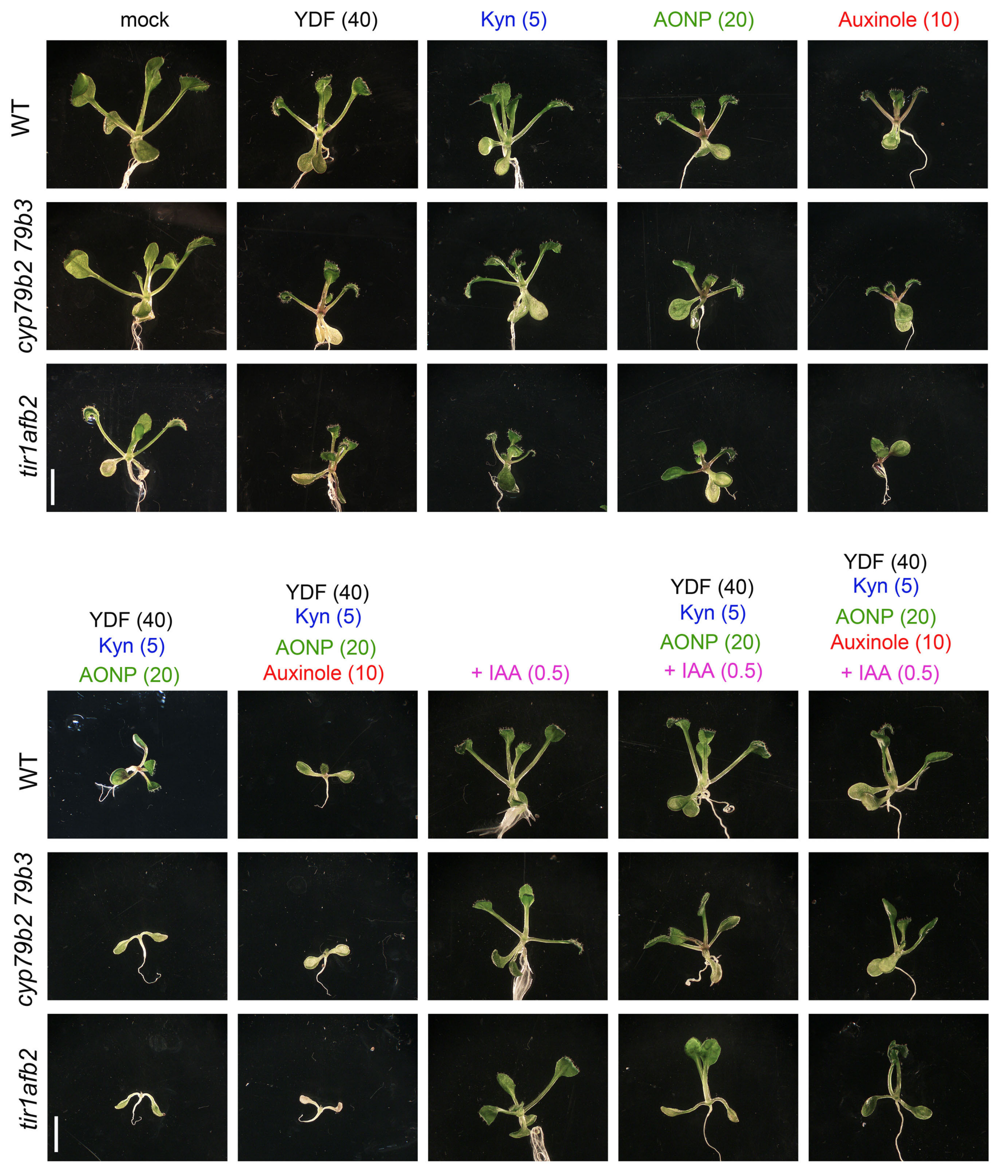

Supplemental Figure S7. Effects of yucasin DF on the phenotypes of Arabidopsis auxin biosynthesis and signaling mutants.

Pictures of representative shoot phenotypes in Fig. 5C. 11-d-old *Arabidopsis* seedlings grown with TAA inhibitors [L-kynurenine (Kyn) and AONP], YUC inhibitor [YDF] and TIR1 antagonist [auxinole] with or without IAA. The values in parentheses indicate concentration (μM). Scale bar = 5 mm.

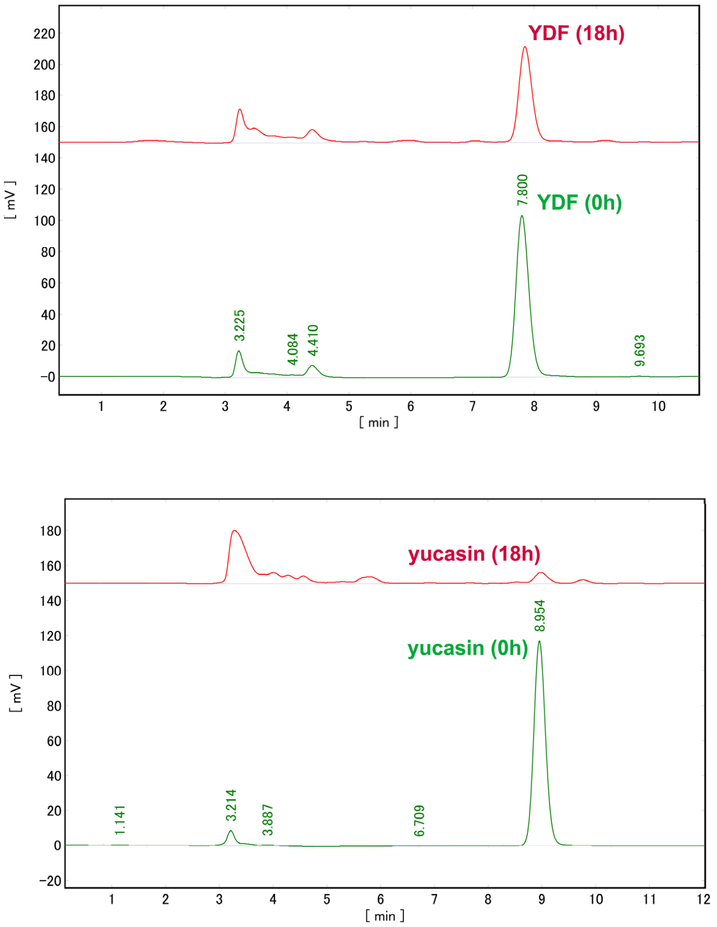

Supplemental Figure S8. HPLC chromatogram of yucasin and YDF after incubation in root cell lysate.

Arabidopsis roots (50 mg) were homogenized in 1.5 mL of 100 mM phosphate buffer (pH 7.0). Inhibitors (250 µM) were added to the cell lysate and incubated for 18 h at 24 °C in the dark. Methanol was added to the lysate (4:1=MeOH:lysate) and centrifuged. The amount of inhibitors were analyzed using HPLC (Cosmosil ODS II column, 4.6 mm × 150 mm; 0.5 mL/min; UV 255 nm detection wavelength; MeOH:H_2O =6:4 + 0.1 % AcOH for the analysis of yucasin, MeOH:H_2O=4:6 + 0.1 % AcOH for the analysis of YDF).

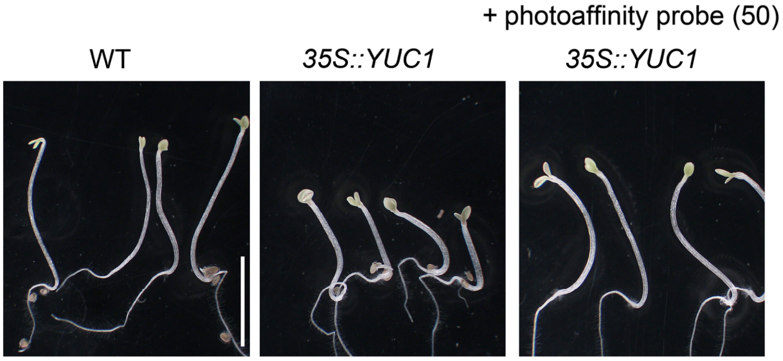

Supplemental Figure S9. Effects of the probe on high-auxin phenotype of *35S::YUC1* etiolated seedlings.

The etiolated seedlings were grown in 1/2 MS medium in the presence of photoaffinity probe at 50 μM in the dark for 3 days. Scale bar = 5 mm.

Supplementary methods

Synthesis of chemicals

General experimental condition.

^{1}H and ^{13}C-NMR spectra were recorded on a JEOL ECS400 (JEOL, Japan). Chemical shifts are shown as δ values from TMS as the internal reference. Peak multiplicities are quoted in Hz. Mass spectra were measured on a JMS-700 spectrometer (JEOL, Japan). Column chromatography was carried out on columns of silica gel 60 (230–400 mesh, Merck, Japan). Reagents used in this study were commercial available chemicals in Japan unless otherwise stated.

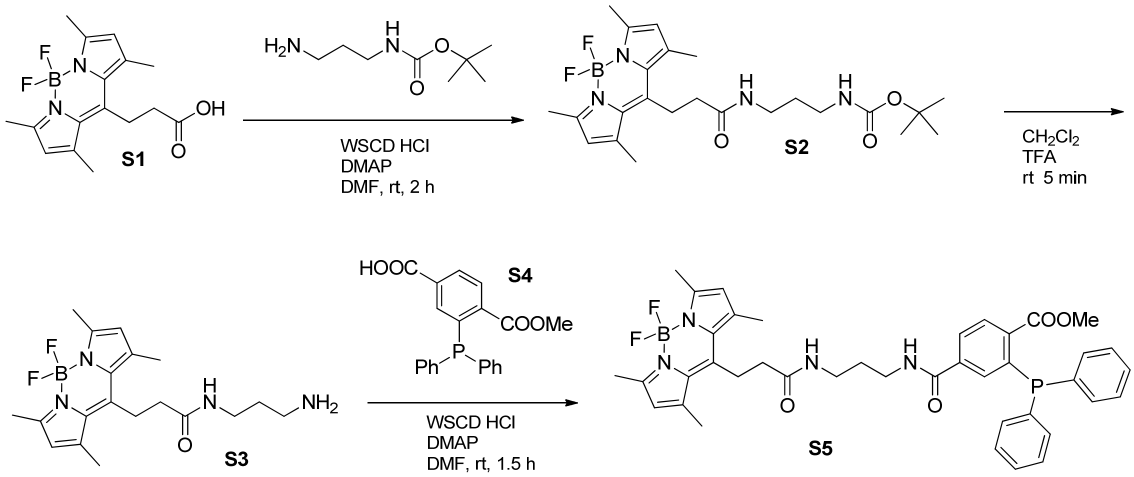

Synthesis of 10-(2-carboxyethyl)-5,5-difluoro- 1,3,7,9-tetramethyl-5H-dipyrrolo [1,2-c:2',1'-f] [1,3,2] diazaborinin-4-ium-5-uide (**S2**)

Carboxyl BODIPY dyes (**S1**, 35.3 mg, 0.11 mmol)[1] and N-(tert-Butoxycarbonyl)-1,3-diaminopropane (38.4mg 0.22mmol) were dissolved in DMF (5 mL) and then 1-(3-dimethylaminopropyl)-3-ethylcarbodiimide hydrochloride (WSCD HCl, 42.3 mg, 0.22 mmol) and 4-dimethylaminopyridine (26.9 mg, 0.22 mmol) was added. The reaction mixture was stirred at room temperature for 2 h. The reaction mixture was added to water (20 mL) and extracted with EtOAc (20 mL X 2). The organic layer was washed with brine and concentrated. The crude product was purified with a silica gel column chromatography (chloroform : acetone = 9 : 1) to afford BODIPY dye with *tert*-BOC-C3-linker (**S2**) as dark orange crystal (22.6 mg, yield

43 %). m.p.= 183 °C, ^{1}H-NMR (400MHz,$CDCl_3$) δ_H 1.43 (s, 9H), 1.56-1.62 (m, 2H), 2.46-2.51 (m, 14H), 3.14 (dd, *J*=5.6, 6.0, 2H), 3.27−3.36 (m, 4H), 6.06 (s, 2H); ^{13}C-NMR (100MHz, $CDCl_3$) δ_C 14.46, 16.47, 24.06, 28.32, 29.23, 29.65, 30.05, 31.39, 35.82, 36.43, 36.96, 37.59, 79.50, 121.79, 131.27, 140.61, 144.52, 154.35, 162.50, 170.78. FAB-MS *m/z* 499 $[M+Na]^+$

Ref. 1 D. Wang, et al. *J. Org. Chem*., 74, 7675-7683; 2009

Synthesis of 10-(3-((3-(3-(diphenylphosphino)-4-(methoxycarbonyl)benzamido) propyl)amino)-3-oxopropyl)-5,5-difluoro-1,3,7,9-tetramethyl-5H-dipyrrolo[1,2-c:2',1'-f][1,3,2]diazaborinin-4-ium-5-uide (**S5**)

BODIPY dye with *tert*-BOC-C3-linker (**S2,** 22 mg 0.05mml) was dissolved in TFA (0.5 mL) and CH_2Cl_2 (1 mL) and stand for 5 min at room temperature. The reaction solution was poured into aqueous sodium bicarbonate (2M, 20 mL) and then extracted with EtOAc (20 mL). The organic layer was washed with brine and concentrated in vacuo. The BODIPY amine (**S3**) was used for next reaction without purification. The BODIPY amine (S3, 18.6 mg, ca 0.05 mmol) and triphenyl phosphine carboxylic acid (**S4**)[2] (25.2 mg 0.07 mmol) were dissolved in DMF (5 mL) and then 1-(3-dimethylaminopropyl)-3-ethylcarbodiimide hydrochloride (WSCD HCl, 18 mg, 0.1 mmol) and 4-dimethylaminopyridine (12 mg, 0.1 mmol) was added. The reaction mixture was stirred at room temperature for 1.5 h. The reaction mixture was added to water (20 mL) and extracted with EtOAc (20 mL X 2). The organic layer was washed with brine and concentrated. The crude product was purified with a silica gel column chromatography (chloroform : acetone＝5 : 1) to afford BODIPY linked phosphine ligand (**S5**) as dark orange crystal (8.0 mg, yield 24.0 % for two steps): m.p.= 166 °C, ^{1}H-NMR (400MHz,$CDCl_3$) δH 1.58−1.62 (m, 2H), 2.43-2.50 (m, 14H), 3.20 (dd, *J*=6.4, 6.4, 2H), 3.25−3.34 (m, 4H), 3.75 (s, 3H), 6.04 (s, 2H), 6.27 (t, *J*=6.2, 1H), 6.78 (t, *J*=6.0, 1H), 7.28−7.36 (m, 11H), 7.79 (dd, *J*=1.2, 2.0, 1H), 8.09 (dd, *J*=3.6, 3.2, 1H); ^{13}C-NMR (100MHz, $CDCl_3$) δ_C 14.46, 16.49, 23.88, 29.54, 29.68, 35.94, 36.03, 37.64, 52.26, 121.92, 126.52, 128.57, 128.65, 128.94, 130.93, 131.28, 132.71, 133.82, 134.03, 137.15, 137.24, 140.50, 144.21, 154.52, 166.64, 166.87, 171.29; FAB-MS *m/z* 723 $[M+H]^+$.

Ref 2: E. Saxon, E.and C.R. Bertozzi, *Science* 287, 2007-2010 (2000).

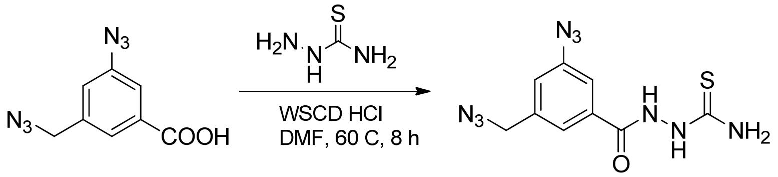

3-azido-5-(azidomethyl)benzoic acid was prepared according to the published procedure[3] 3-azido-5-(azidomethyl)benzoic acid (170 mg, 0.78 mmol) and thiosemicarbazide (213 mg, 2.34 mmol) were dissolved in DMF (5 mL) and then 1-(3-dimethylaminopropyl)- 3-ethylcarbodiimide hydrochloride (WSCD HCl, 2.4 mmol) was added. The solution was stirred at 60 °C for 8 h. The reaction mixture was poured into water (50 mL) then acidified to pH 3-4 with 2M HCl. The mixture was extracted with EtOAc (40 mL) twice and concentrated in vacuo. The mixture was suspended in water and the resulting precipitate was collected by filtration and washed with distilled water. The product was dried in vacuo to afford 2-(3-azido-5-(azidomethyl)benzoyl)hydrazinecarbothioamide as amorphous powder (74 mg, yield 33%). ^{1}H-NMR (400MHz, DMSO-d_6) δ_H 4.55 (s, 2H), 7.32 (t, *J*=1.6 Hz, 1H), 7.65 (t, *J*=1.5, 1H), 7.69 (t, *J*=1.5Hz, 1H), 9.38 (s, 1H), 10.54 (s, 1H); ^{13}C-NMR (100MHz, DMSO-d_6) δ_C 52.72, 118.05, 121.89, 124.53, 134.67, 138.09, 139.94, 164.69, 182.01; FAB-MS *m/z* 292 $[M+H]^+$.

Ref. 3: R. Neelarapu, et. al., J. Med. Chem. 54, 13, 4350-4364 (2011).

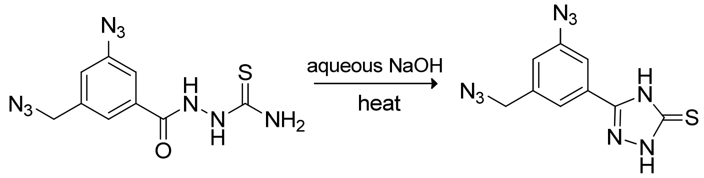

2-(3-azido-5-(azidomethyl)benzoyl)hydrazinecarbothioamide (50 mg, 0.18 mmol) was suspended in aqueous sodium hydroxide (3 equivalent of NaOH in 10 mL water) and stirred at 80 °C for 3 h. The resulting mixture was added to water (50 mL) then acidified with 2M HCl. The precipitate was collected by filtration and washed with distilled water. The product was dried in vacuo to afford 3-(3-azido-5-(azidomethyl)phenyl)-1H-1,2,4-triazole-5(4H)-thione as amorphous powder (34 mg, yield 62 %) . ^{1}H-NMR (400MHz, DMSO-d_6) δ_H 4.57 (s, 2H), 7.24 (s, 1H), 7.66 (t, J = 1.8, 1H), 7. 75 (s, 1H); ^{13}C-NMR (100MHz, DMSO-d_6) δ_C 53.2, 116.2, 121.2, 122.2, 127.9, 139.6, 141.5, 149.7, 167.8; FAB-MS *m/z* 274 [M+H]+.

Compound **1**: Yucasin DF

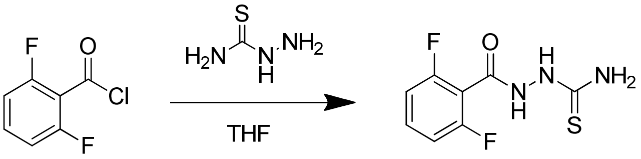

Intermediate of compound **1** (YDF), Amorphous powder, m.p. >180-189 °C, yield 63 % (method B), ^{1}H-NMR (400MHz, DMSO-d_6); 6.98 (1H, s), 7.21 (4H, t, *J*=8.4Hz), 7.59 (2H, m), 8.15 (1H, s), 9.75 (2H, s), 10.61 (2H, s), ^{13}C-NMR (100 MHz, DMSO-d_6); δ_C 112.1, 112.2, 112.5, 133.0, 158.2, 158.3, 160.7, 160.8, 182.0, 182.1; FAB-MS *m/z* 232[M+H]$^+$.

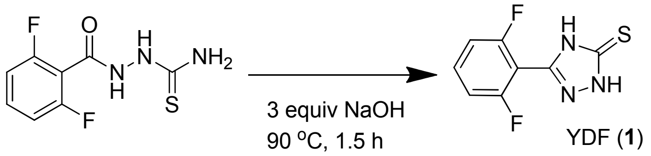

Yucasin DF (YDF, **1**): Amorphous powder, m.p. >200 °C, yield 82 %, ^{1}H-NMR (400MHz, DMSO-d_6) 7.35 (2H, t, *J*=8.8Hz), 7.70 (1H, m), ^{13}C-NMR (100 MHz, DMSO-d_6) δ_C 104.2, 112.6, 112.8, 134.0, 134.1, 134.2, 158.8, 161.3, 167.2; FAB-MS *m/z* 214[M+H]$^+$.

Yucasin (**2**) is commercially available compound [CAS: 26028-65-9] from Sigma-Aldrich (Japan) and WAKO pure chemicals (Japan).

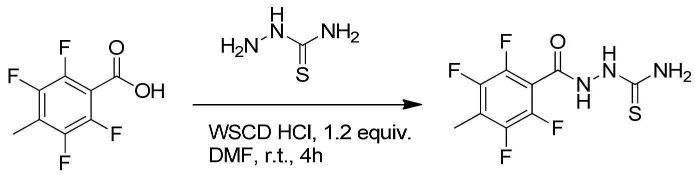

Intermediate of analogs 3, Amorphous powder, m.p. >200 °C, yield 70 % (method A), ^{1}H-NMR (400MHz, DMSO-d_6); δ_H 2.80 (3H, s), 9.72 (1H, s), 10.74 (1H , , s); ^{13}C-NMR (100 MHz, DMSO-d_6); δ_C 7.87, 112.43, 118.65, 141.76,141.93, 143.11, 143.27, 144.24, 144.46, 145.65, 145.74, 157.36, 181.45; FAB-MS *m/z* 282[M+H]$^+$.

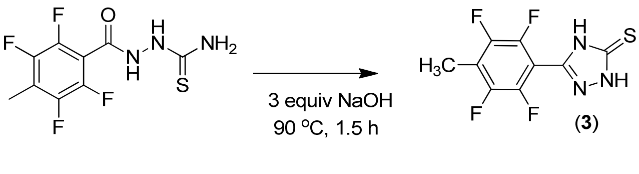

Amorphous powder, m.p. >200 °C, yield 84 % (method A), ^{1}H-NMR (400MHz, DMSO-d_6); δH 2.27 (3H, s); ^{13}C-NMR (100 MHz, DMSO-d_6); δC 8.38, 103.82, 103.98, 112.36, 112.73, 120.22, 120.42, 120.62, 140.07, 142.73, 142.79, 142.88, 145.19, 145.35, 146.38, 146.61, 167.82; FAB-MS *m/z* 264$[M+H]^+$.

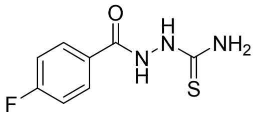

Intermediate of **4**, Amorphous powder, m.p. 189-200 °C, yield 65 % (method B), ^{1}H-NMR (400MHz, acetone-d_6); 7.27 (2H, t, J=8.8 Hz), 8.05 (2H, t, J=8.8 Hz), ^{13}C-NMR (100 MHz, acetone-d_6); δ_C 115.5 (115.5, 115.6: d, $J_{C\text{-}F}$=21.9 Hz), 129.3, 130.7, 130.8 (d, $J_{C\text{-}F}$ =9.5 Hz), 164.0, 165.5 (d, $J_{C\text{-}F}$ =96.3 Hz), 184.8; FAB-MS *m/z* $[M+H]^+$.

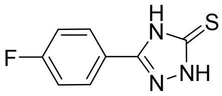

4

Compound **4**: Amorphous powder, m.p. >200 °C, yield 90 %, ^{1}H-NMR (400MHz, DMSO-d_6); δH 7.33 (2H, tt, J_{HF}=2.4, 7.1 Hz), 8.05 (2H, tt, J_{HF} =2.4, 5.9 Hz), ^{13}C-NMR (100 MHz, DMSO-d_6); 116.4 (116.4, 116.5: $J_{C\text{-}F}$=17.7 Hz), 122.6, (d, $J_{C\text{-}F}$ =2.5 Hz), 128.6 (128.6, 128.7: d, $J_{C\text{-}F}$ =7.0 Hz), 150.0, 163.4, 196.1; FAB-MS *m/z* 196 $[M+H]^+$.

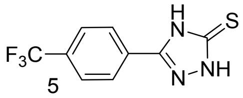

5

Known compound (**5**), 5-(4'-fluorophenyl)- 2H-1,2,4-triazole- 3(4H)-thione synthesized by method A was agreed with published data in following article: J. Cui et al., *ChemMedChem*, 11, 1, 43-56 (2016).

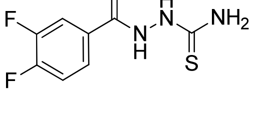

Intermediate of compound **6**, Amorphous powder, m.p. >200 °C, yield 75 % (method A), ^{1}H-NMR (400MHz, acetone-d_6); 7.44-7.53 (1H, m), 7.86-7.982 (2H, m), 8.77 (1H, s), 10.01 (1H, s), ^{13}C-NMR (100 MHz, acetone-d_6) δ_C 118.24, 118.33, 118.43, 118.48,

125.97, 130.67, 149.53, 149.64, 151.50, 151.60, 152.22, 152.32, 154.22, 154.33, 165.12; FAB-MS *m/z* 232 $[M+H]^+$.

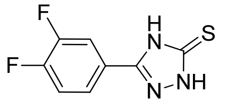

Compound **6**: Amorphous powder, m.p. >200 °C, yield 49 %, ^{1}H-NMR (400MHz, acetone-d_6); 7.49-7.58 (1H, m), 7.82-7.88 (1H, m), 7.90-7.97 (1H, m), 12.50 (1H, s), 12.69 (1H, s); ^{13}C-NMR (100 MHz, acetone-d_6) δ_C 115.47, 115.62, 118.68, 118.82, 123.35, 123.38, 123.41, 123.44, 149.66, 149.77, 150.79, 150.89, 151.63, 151.74, 152.79, 169.42; FAB-MS *m/z* 214 $[M+H]^+$.

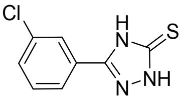

7

Known compound, 5-(3’-chlorophenyl)-2H-1,2,4-triazole-3(4H)-thione (**7**) synthesized by method A was agreed with published data in following article: I.M. Westwood et al., *Protein & Cell*, 1, 1, 82-95 (2010)

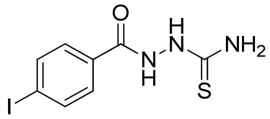

Intermediate of compound **8**, Amorphous powder, m.p. >200 °C, yield 69 % (method A), ^{1}H-NMR (400MHz, DMSO-d_6); δ_H 7.67 (2H, d, *J*=8.4 Hz), 7.88 (2H, d, *J*=8.6 Hz), 9.35 (1H, s), 10.45 (1H, s); ^{13}C-NMR (100 MHz, DMSO-d_6) δ_C 99.8, 130.0, 132.2, 137.3, 165.6; FAB-MS *m/z* 321 $[M+H]^+$.

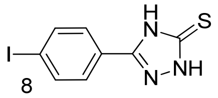

8

Compound **8**: Amorphous powder, m.p. >200 °C, yield 84 %, ^{1}H-NMR (400MHz, DMSO-d_6) δ_H 7.71 (2H, d, *J*=8.6 Hz), 7.92 (2H, d, *J*=8.6 Hz), 13.76 (1H, s); ^{13}C-NMR (100 MHz, DMSO-d_6) δ_C 97.9, 125.1, 127.7, 138.1, 149.8, 167.4; FAB-MS *m/z* 304$[M+H]^+$.

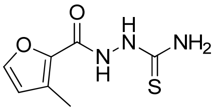

Intermediate of compound **9**, Amorphous powder, m.p. >200 °C, yield 23 % (method A), ^{1}H-NMR (400MHz, DMSO-d_6) δ_H 2.26 (3H, s), 6.51 (1H, d, *J*=1.6), 7.69 (1H, d, *J*=1.6), 9.20 (1H, s), 10.05 (1H, s); ^{13}C-NMR (100 MHz, DMSO-d_6) δ_C 11.10, 115.39, 127.98, 141.19, 143.87, 158.55, 182.32; FAB-MS *m/z* 200 $[M+H]^+$.

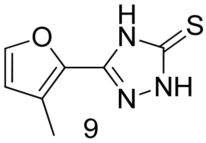

9

Compound **9**: Amorphous powder, m.p. >200 °C, yield 44 %, ^{1}H-NMR (400MHz, DMSO-d_6) δ_H 2.21 (3H, s), 6.57 (1H, d, *J*=1.6), 7.77 (1H, d, *J*=1.6), 13.67 (1H, s), 13.76 (1H, s); ^{13}C-NMR (100 MHz, DMSO-d_6) δ_C 10.92, 115.60, 123.00, 136.79, 144.24, 144.74, 166.64; FAB-MS *m/z* 182 $[M+H]^+$.

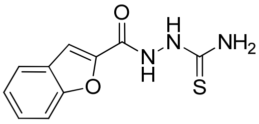

Intermediate of compound **10**, Amorphous powder, m.p. >200 °C, yield 55 % (method A), ^{1}H-NMR (400MHz, DMSO-d_6) δ_H 7.36 (1H, ddd, *J*=7.0, 7.1 Hz), 7.50 (1H, ddd, *J*=8.4, 8.2 Hz), 7.67 (1H, s), 7.69 (1H, dd, *J*=8.4 Hz), 7.82 (1H, dd, *J*=7.3 Hz), 9.48 (1H, s), 10.71 (1H, s); ^{13}C-NMR (100 MHz, DMSO-d_6) δ_C 110.8, 112.1, 123.1, 124.1, 127.1, 127.4, 147.9, 154.9, 158.2, 182.4; FAB-MS *m/z* 236 $[M+H]^+$.

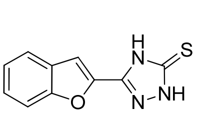

10

Compound **10**: Amorphous powder, m.p. >200 °C, yield 86 %, ^{1}H-NMR (400MHz, DMSO-d_6) δ_H 7.38 (1H, ddd, *J*=7.5, 7.5 Hz), 7.48 (1H, ddd, *J*=7.3, 8.3 Hz), 7.63 (1H, d, *J*=0.7 Hz), 7.73 (1H, dd, *J*=8.3 Hz), 7.82 (1H, dd, *J*=7.1 Hz), 13.96 (1H, s), 14.24 (1H, s); ^{13}C-NMR (100 MHz, DMSO-d_6) δ_C 108.0, 111.8, 122.5, 124.2, 126.7, 127.4, 142.4,

143.3, 154.7, 167.4; FAB-MS *m/z* 218 $[M+H]^+$.

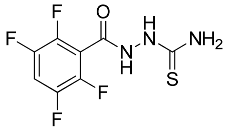

Intermediate of compound **11**, Amorphous powder, m.p. >200 °C, yield 95 % (method A), ^{1}H-NMR (400MHz, DMSO-d_6) δ_H 7.37 (1H, s), 9.78 (1H, s), 10.80 (1H, s); ^{13}C-NMR (100 MHz, DMSO-d_6) δ_C 108.41, 108.65, 108.87, 115.26, 115.48, 115.67, 141.79, 141.95, 143.95, 144.05, 144.19, 144.29, 144.44, 146.36, 146.50, 157.08, 162.41, 181.99; FAB-MS *m/z* 268 $[M+H]^+$.

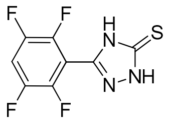

Compound **11**: Amorphous powder, m.p. >200 °C, yield 28 %, ^{1}H-NMR (400MHz, DMSO-d_6) δ_H 8.07-8.16 (1H, m), 14.19 (1H, s); ^{13}C-NMR (100 MHz, DMSO-d_6); δ_C 107.00, 107.12, 107.24, 109.64, 109.78, 109.96, 110.15, 139.69, 139.85, 143.07, 143.10, 143.13, 143.19, 143.22, 144.96, 145.00, 145.05, 145.09, 145.15, 145.19, 145.23, 145.26, 146.92, 146.96, 147.01, 147.12, 147.15, 167.85; FAB-MS *m/z* 250 $[M+H]^+$.

12 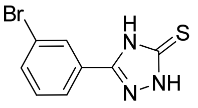

Known compound (**12**) 3-(3-bromophenyl)-1H- 1,2,4-triazole- 5(4H)-thione synthesized by method A was agreed with published data in following article: W.Y. Liu., et. al.,Spectrochimica Acta, Part A: *Molecular and Biomolecular Spectroscopy*, 76A, 5, 531-536 (2010)

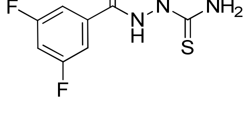

Intermediate of compound **13**, Amorphous powder, m.p. >200 °C, yield 60 % (method A), ^{1}H-NMR (400MHz, acetone-d_6) δ_H 7.24 (1H, t, J_{HF}=8.5 Hz), 7.61 (2H, d, J_{HF}=6.4 Hz); ^{13}C-NMR (100 MHz, acetone-d_6) δ_C 107.3, 107.6, (107.6, 107.8, t, $J_{C\text{-}F}$=25.8 Hz),

111.7 (111.6, 111.9, d, $J_{C\text{-}F}$=26.7 Hz), 137.6 (s), 162.4 (162.3, 162,5, d, $J_{C\text{-}F}$=12.4 Hz), 164.2 (s), 164.9 (164.8, 164.9, d, $J_{C\text{-}F}$=12.4 Hz) ; FAB-MS *m/z* 232 $[M+H]^+$.

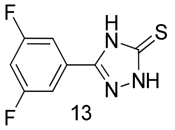

13

Compound **13**: Amorphous powder, m.p. >200 °C, yield 95 %, ^{1}H-NMR (400MHz, acetone-d_6) δ_H 7.24 (1H, tt, J_{HF}=2.4, 9.2 Hz), 7.62 (2H, m); ^{13}C-NMR (100 MHz, acetone-d_6)d δ_C 106.0, 106.2, 106.5 (106.2, t, $J_{C\text{-}F}$=15.7 Hz), 109.4, 109.5, 109.6, 109.7 (109.5, dd, $J_{C\text{-}F}$=8.5, 8.6 Hz), 129.5(129.4, 129.5, t, $J_{C\text{-}F}$=10.5 Hz), 149.0 (s), 162.5, 162.6, 165.0, 169.6; FAB-MS *m/z* 214 $[M+H]^+$.

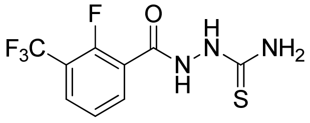

Intermediate of compound **14**, Amorphous powder, m.p. >200 °C, yield 61 % (method A), ^{1}H-NMR (400MHz, DMSO-d_6) δ_H 7.46 (1H, t, *J*=7.8), 7.87 (1H, t, *J*=7.0), 8.01 (1H, t, J=6.96), 9.48 (1H, s), 10.48 (1H, s); ^{13}C-NMR (100 MHz, DMSO-d_6); δ_C 117.96, 118.08, 118.28, 118.40, 121.80, 124.51, 125.75, 130.69, 135.82, 156.02, 163.42, 182.57; FAB-MS *m/z* 282 $[M+H]^+$.

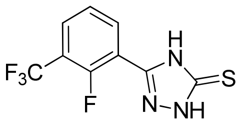

Compound **14**: Amorphous powder, m.p. >200 °C, yield 85 %, ^{1}H-NMR (400MHz, DMSO-d_6) δH 7.60 (1H, t, *J*=7.8), 7.96 (1H, t, *J*=6.9), 8.18 (1H, t, *J*=6.8), 13.98 (1H, s); ^{13}C-NMR (100 MHz, DMSO-d6) δ_C 115.53, 115.65, 117.70, 117.81, 118.02, 118.14, 118.34, 121.03, 123.74, 125.51, 125.55, 129.39, 134.15, 154.85, 157.48, 167.27; FAB-MS *m/z* 264 $[M+H]^+$.

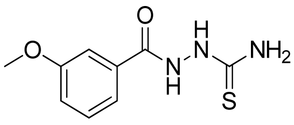

Intermediate of compound **15**, Amorphous powder, m.p. >200 °C, yield 60 % (method A), ^{1}H-NMR (400MHz, DMSO-d$_6$) δ_H 3.81 (3H, s), 7.13 (1H, d, *J*=8.2 Hz), 7.38 (1H, t, *J*=8.2 Hz), 7.48 (2H, d, *J*=6.8 Hz), 7.65 (1H, s), 7.90 (1H, s), 9.36 (1H, s), 10.39 (1H, s); ^{13}C-NMR (100 MHz, DMSO-d$_6$); δ_C 55.6, 113.2, 117.9, 120.3, 129.6, 134.1, 159.2, 165.9, 182.1; FAB-MS *m/z* 226 [M+H]$^+$.

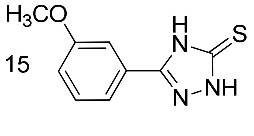

15

Compound **15**: Amorphous powder, m.p. >200 °C, yield 90 %, ^{1}H-NMR (400MHz, DMSO-d$_6$) δ_H 3.76 (3H, s), 6.99 (1H, d, *J*=8.1 Hz), 7.36 (1H, t, *J*=8.1 Hz), 7.46 (2H, d, *J*=7.9 Hz) 13.67 (1H, s), 13.79 (1H, s); ^{13}C-NMR (100 MHz, DMSO-d$_6$) δ_C 55.6, 111.0, 116.9, 118.2, 126.9, 130.6, 150.5, 159.9, 167.3; FAB-MS *m/z* 208 [M+H]$^+$.

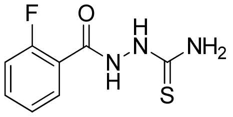

Intermediate of yucasin monofluorinated, YMF (**16**), Amorphous powder, m.p. >200 °C, yield 57 % (method A), ^{1}H-NMR (400MHz, DMSO-d$_6$) δ_H 7.30 (2H, t, *J*=8.64), 7.54-7.61 (1H, m), 7.82 (1H, t, *J*=7.32), 7.98 (1H, s), 9.48 (1H, s), 10.23 (1H, s); ^{13}C-NMR (100 MHz, DMSO-d$_6$) δ_C 116.31, 116.49, 121.99, 122.09, 124.51, 130.90, 133.38, 133.44, 158.76, 163.68, 182.15; FAB-MS *m/z* 214 [M+H]$^+$.

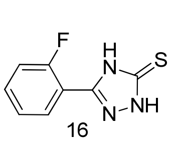

16

Yucasin monofluorinated, YMF (**16**): Amorphous powder, m.p. >200 °C, yield 78 %, ^{1}H-NMR (400MHz, DMSO-d$_6$) δ_H 7.35-7.46 (2H, m), 7.57-7.65 (1H, m), 7.82-7.88 (1H, m), 13.78 (1H, s), 13.86 (1H, s); ^{13}C-NMR (100 MHz, DMSO-d$_6$) δ_C 113.81, 113.91, 116.57, 116.74, 125.01, 125.04, 129.48, 129.49, 132.76, 132.83, 146.21, 146.23, 158.12, 166.95; FAB-MS *m/z* 196 [M+H]$^+$.

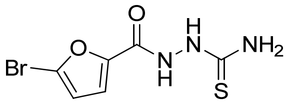

Intermediate of **17**, Amorphous powder, m.p. >200 °C, yield 57 % (method A), ^{1}H-NMR (400 MHz, DMSO-d$_6$); δ_H 6.78 (1H, d, *J*=3.7 Hz), 7.23 (1H, d, *J*=3.3 Hz), 9.33 (1H, s), 10.38 (1H, s); ^{13}C-NMR (100 MHz, DMSO-d$_6$) δ_C 114.2, 117.3, 125.4, 148.4, 162.6, 182.3; FAB-MS *m/z* 264 $[M+H]^+$.

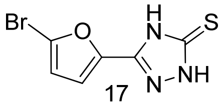

17

Compound **17**: Amorphous powder, m.p. >200 °C, yield 92 %, ^{1}H-NMR (400MHz, DMSO-d$_6$) δ_H 6.84 (1H, d, *J*=3.2 Hz), 7.15 (1H, d, *J*=3.6 Hz); ^{13}C-NMR (100 MHz, DMSO-d$_6$) δ_C 114.4, 114.6, 125.0, 142.5, 142.6, 166.9; FAB-MS *m/z* 246 $[M+H]^+$.

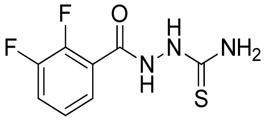

Intermediate of **18**, Amorphous powder, m.p. >200 °C, yield 80 % (method A), ^{1}H-NMR (400 MHz, DMSO-d$_6$) δ_H 7.29-7.35 (1H, m), 7.57-7.65 (3H, m), 8.01 (1H, s), 9.51 (1H, s), 10.38 (1H, s) ^{13}C-NMR (100 MHz, DMSO-d$_6$) δ_C 119.92, 120.06, 124.15, 124.77, 125.40, 125.63, 148.74, 148.83, 150.69, 150.78, 162.40, 181.95; FAB-MS *m/z* 232 $[M+H]^+$.

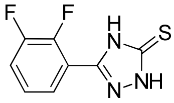

18

Compound **18**: Amorphous powder, m.p. >200 °C, yield 77 %, ^{1}H-NMR (400MHz, DMSO-d$_6$) δ_H 7.36-7.43 (1H, m) , , 7.59-7.71 (2H, m) , , 13.95 (2H, s); ^{13}C-NMR (100 MHz, DMSO-d$_6$); δ_C 119.73, 119.87, 124.60, 124.62, 125.66, 125.69, 125.71, 125.75, 146.41, 146.52, 148.45, 148.56, 149.40, 149.49, 151.36, 151.45, 167.35; FAB-MS *m/z* 214 $[M+H]^+$.

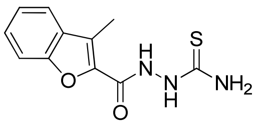

Intermediate of **19**, Amorphous powder, m.p. >200 °C, yield 53 % (method A), ^{1}H-NMR (400 MHz, DMSO-d_6) δ_H 2.55 (3H, s) 7.36 (1H, t, *J*=7.2 Hz), 7.50 (1H, t, *J*=6.8 Hz), 7.59 (1H, d, *J*=8.4 Hz), 7.77 (1H, d, *J*=4 Hz), 9.36 (1H, s), 10.49(1H, s); ^{13}C-NMR (100 MHz, DMSO-d_6): δ_C 9.0, 111.8, 121.3, 122.5, 123.5, 127.7, 129.1, 142.2, 153.0, 159.2, 182.3; FAB-MS *m/z* 250 $[M+H]^+$.

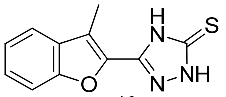

19

Compound **19**: Amorphous powder, m.p. >200 °C, yield 92 %, ^{1}H-NMR (400MHz, DMSO-d_6) δ_H 2.49 (3H, s), 7.37 (1H, t, *J*=7.6 Hz), 7.46 (1H, t, *J*=7.2 Hz), 7.62(1H, d, *J*=8.4Hz), 7.74 (1H, d, *J*=7.6Hz); ^{13}C-NMR (100 MHz, DMSO-d_6): δ_C 8.6, 111.6, 117.5, 120.8, 123.7, 126.6, 129.2, 138.0, 143.9, 153.8, 166.9; FAB-MS *m/z* 232 $[M+H]^+$.

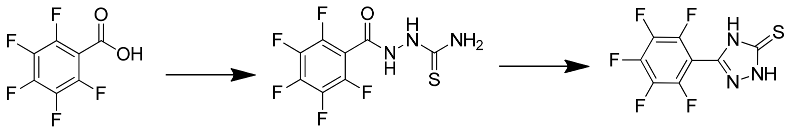

Intermediate of **20**, Amorphous powder, m.p. >200 °C, yield 53 % (method A). This intermediate was used for next reaction. Compound **22**: Amorphous brown powder, m.p. >200 °C, yield 44 %, ^{13}C-NMR (100 MHz, DMSO-d_6); δ_C 133.5, 139.5, 139.6, 139.9, 142.1, 143.5, 143.7, 145.5, 145.9, 146.1, 148.0, 148.1, 167.3; FAB-MS *m/z* 268 $[M+H]^+$.

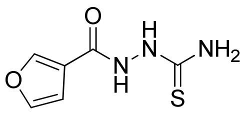

Intermediate of **21**, Amorphous powder, m.p. >200 °C, yield 53 % (method A), ^{1}H-NMR (400 MHz, DMSO-d_6) δ_H 6.86 (1H, s), 7.61 (1H, s), 7.75 (2H, t, *J*=1.7 Hz), 7.88 (1H, s), 8.25 (1H, s), 9.33 (1H, s), 10.13 (1H, s); ^{13}C-NMR (100 MHz, DMSO-d_6) δ_C 109.4, 120.9, 144.2, 146.1, 161.5, 182.4; FAB-MS *m/z* 186 $[M+H]^+$.

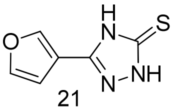

21

Compound **21**: Amorphous powder, m.p. >200 °C, yield 35 %, ^{1}H-NMR (400MHz, acetone-d_6) δ_H 6.93 (1H, dd, *J*=0.7, 0.9 Hz), 7.76 (1H, t, *J*=1.7 Hz), 8.26 (1H, dd, *J*=0.7, 0.9 Hz), 12.48 (1H, d, *J*=44.9 Hz); ^{13}C-NMR (100 MHz, acetone-d_6) δ_C 108.3, 113.6, 142.8, 145.1, 145.2, 168.5; FAB-MS *m/z* 168 $[M+H]^+$.

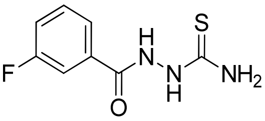

Intermediate of **22**, Amorphous powder, m.p. 189-193 °C, yield 75 % (method A), ^{1}H-NMR (400 MHz, DMSO-d_6) 7.37-7.42 (1H, m), 7.48-7.55 (1H, m), 7.69-7.73 (3H, m), 7.93 (1H, s), 9.37 (1H, s), 10.47 (1H, s); ^{13}C-NMR (100MHz, DMSO-d_6) δ_C 111.78, 111.97, 114.84, 115.03, 118.77, 118.94, 124.26, 130.60, 130.66, 135.05, 135.11, 164.83, 182.28; FAB-MS *m/z* 214 $[M+H]^+$.

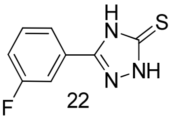

22

Compound **22**: Amorphous powder, m.p. >200 °C, yield 54 %, ^{1}H-NMR (400MHz, DMSO-d_6) δ_H 7.35-7.41 (1H, m), 7.56-7.64 (1H, m), 7.73-7.82 (2H, m), 13.83 (1H, s), 13.97 (1H, s); 13C-NMR (100 MHz, DMSO-d_6) δ_C 112.62, 112.08, 117.55, 117.72, 122.00, 122.03, 127.75, 127.82, 131.55, 131.61, 149.36, 149.38, 161.52, 167.50; FAB-MS *m/z* 196 $[M+H]^+$.

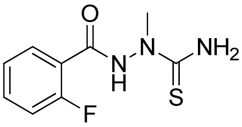

Intermediate of **23**, Amorphous powder, m.p. >200°C, yield 51 % (method A), ^{1}H-NMR (400 MHz, DMSO-d_6) δ_H 3.45 (1H, s), 7.31 (1H, m), 7.34 (1H, m), 7.60 (1H, m), 7.84 (1H, m),7.95 (1H, s), 10.60 (1H, s); ^{13}C-NMR (100 MHz, DMSO- d_6); δ_C 40.7, 116.3,

116.5, 121.5, 121.7, 124.6, 130.9, 133.6, 133.7, 158.6, 161.1, 182.6; FAB-MS *m/z* 228 $[M+H]^+$.

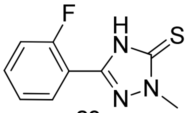

23

Compound **23**: Amorphous powder, m.p. >200 °C, yield 97 %, ^{1}H-NMR (400MHz, DMSO-d_6) δ_H 3.19 (1H, s), 3.73 (3H, s), 7.38 (1H, m), 7.43 (1H, m), 7.62 (2H, m),7.82 (1H, ddd, *J*=7.2,7.2,1.2Hz); ^{13}C-NMR (100 MHz, DMSO-d_6) δ_C 35.6, 113.7, 113.8, 116.9, 117.1, 125.4, 129.7, 133.3, 158.1, 160.4, 166.2; FAB-MS *m/z* 210 $[M+H]^+$.

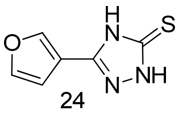

24

Compound **24** [CAS: 35771-65-4] is commercially available from Sigam Aldrich

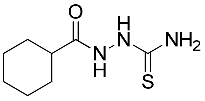

Intermediate of **25**, Amorphous powder, m.p. >200 °C, yield 18 % (method A), ^{1}H-NMR (400 MHz, DMSO-d_6) δ_H 1.23 (9H, m), 1.72 (9H, m), 2.13 (5H, s), 7.31 (1H, s), 9.13 (1H, s), 9.63 (1H, s); ^{13}C-NMR (100 MHz, DMSO-d_6) δ_C 25.5, 25.7, 29.0, 175.0, 182.1; FAB-MS *m/z* 202 $[M+H]^+$.

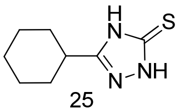

25

Compound **25**: Amorphous powder, m.p. >200 °C, yield 67 %, ^{1}H-NMR (400MHz, DMSO-d_6) δ_H1.18 (1H, m), 1.30 (3H, q, *J*=36.8Hz), 1.42 (3H, q, *J*=38.4Hz), 1.63 (1H, d, *J*=12.4Hz), 1.72 (3H, d, *J*=12.8Hz), 1.87 (3H, d, *J*=10Hz), 2.57 (1H, m); ^{13}C-NMR (100 MHz, DMSO-d_6) δ_C 25.6, 25.7, 30.4, 35.1, 156.6, 166.3; FAB-MS *m/z* 184 $[M+H]^+$.

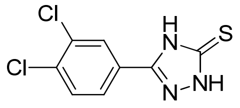

26

Known compound (**26**) , , 3-(3,4-dichlorophenyl)-1H-1,2,4-triazole- 5(4H)-thione synthesized by method A was agreed with published data in following article: I.M. Westwood et al., Protein & Cell, 1, 1, 82-95 (2010)

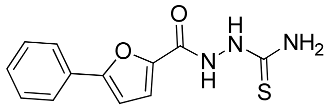

Intermediate of **27**, Amorphous powder, m.p. >200°C, yield 52 % (method A), ^{1}H-NMR (400 MHz, DMSO-d_6) δ_H 7.15 (1H, d, *J*=3.5 Hz), 7.32 (1H, d, *J*=3.5 Hz), 7.39 (1H, t, *J*=7.3 Hz), 7.49 (2H, t, *J*=7.1 Hz), 7.96 (2H, d, *J*=7.3 Hz), 9.40 (1H, s), 10.52 (1H, s); ^{13}C-NMR (100 MHz, DMSO-d_6) δ_C 107.8, 117.3, 124.7, 129.0, 129.2, 129.5, 145.8, 155.2; FAB-MS *m/z* 262 $[M+H]^+$.

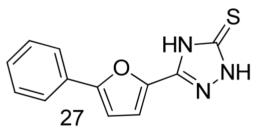

27

Compound **27**: Amorphous powder, m.p. >200 °C, yield 97 %, ^{1}H-NMR (400MHz, DMSO-d_6) δ_H 7.19 (1H, d, *J*=2.7 Hz), 7.27 (1H, d, *J*=3.7 Hz), 7.40 (1H, t, *J*=7.3 Hz), 7.50 (2H, t, *J*=7.5 Hz), 7.89 (2H, d, *J*=7.3 Hz), 13.81 (1H, s); ^{13}C-NMR (100 MHz, DMSO-d_6) δ_C 108.1, 113.8, 124.4, 128.8, 129.3, 129.5, 140.1, 143.4, 155.1, 166.9; FAB-MS *m/z* 244 $[M+H]^+$.

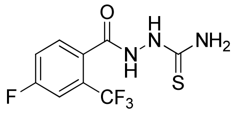

Intermediate of **28**, Amorphous powder, m.p. >171-172 °C, yield 76 % (method A), ^{1}H-NMR (400 MHz, DMSO-d_6) δ_H 7.59 (1H, s), 7.65 (1H, t, *J*=8.3), 7.72 (1H, dd, *J*=2.6, 9.2), 9.54 (1H, s), 10.52 (1H, s); ^{13}C-NMR (100 MHz, DMSO- d_6) δ_C 114.19, 119.06, 119.26, 121.28, 132.56, 161.06, 163.54, 165.61, 181.89; FAB-MS *m/z* 282 $[M+H]^+$.

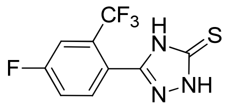

28

Compound **28**: Amorphous powder, m.p. >200 °C, yield 54 %, ^{1}H-NMR (400MHz, DMSO-d$_6$) δ_H 7.40 (1H, s), 7.67 (1H, t, *J*=8.4), 7.75 (1H, dd, *J*=2.6, 9.0), 9.59 (1H, s), 10.44 (1H, s); ^{13}C-NMR (100 MHz, DMSO-d$_6$) δ_C 114.45, 114.69, 119.22, 119.43, 130.25, 132.72, 161.26, 163.74, 165.81, 166.92, 182.09; FAB-MS *m/z* 264 [M+H]$^+$.

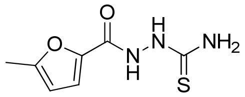

Intermediate of **29**, Amorphous powder, m.p. >200 °C, yield 54 % (method A), ^{1}H-NMR (400 MHz, DMSO-d$_6$) δ_H 2.33 (3H, s), 6.27 (1H, d, *J*=2.4 Hz), 7.10 (1H, d, *J*=2.8 Hz), 9.27 (1H, s), 10.15 (1H, s); ^{13}C-NMR (100 MHz, DMSO-D6): δ_C 13.7, 108.4, 116.1, 144.9, 155.2, 157.6, 182.4; FAB-MS *m/z* 200 [M+H]$^+$.

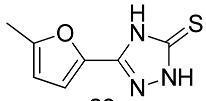

29

Compound **29**: Amorphous powder, m.p. >200 °C, yield 83 %, ^{1}H-NMR (400MHz, DMSO-d$_6$) δ_H 2.35 (3H, s), 6.31 (1H, dd, J=0.8 Hz, 3.2 Hz), 7.03 (1H, d, J=3.2 Hz); ^{13}C-NMR (100 MHz, DMSO-d$_6$) δ_C 13.6, 108.5, 113.3, 139.0, 143.5, 154.9, 166.6; FAB-MS *m/z* 282 [M+H]$^+$.

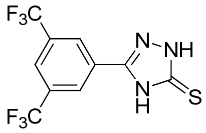

5-[3,5-Bis(trifluoromethyl)phenyl]-1,2,4-triazole-3-(2H)-thione (**30**) [CAS: 175276-77-4] is commercially available from Sigma Aldrich.

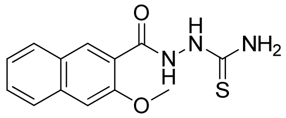

Intermediate of **31**, Amorphous powder, m.p. >200 °C, yield 54 % (method A),

^{1}H-NMR (400 MHz, DMSO-d$_6$) δ_H 3.90 (3H, s), 7.25 (1H, s), 7.37 (1H, t, J=7.3), 7.43 (2H, s), 7.51 (1H, t, *J*=7.3), 7.83 (1H, d, J=8.6), 7.89 (1H, d, *J*=8.6), 8.28 (1H, s), 9.49 (1H, s), 10.14 (1H, s); ^{13}C-NMR (100 MHz, DMSO-d$_6$) δ_C 56.09, 106.69, 124.34, 124.67, 126.75, 127.65, 128.18, 128.56, 130.92, 135.36, 154.43, 165.44, 182.06; FAB-MS *m/z* 276 [M+H]$^+$.

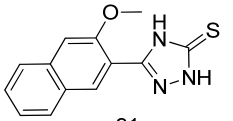

31

Compound **31**, Amorphous powder, m.p. >200 °C, yield 23 %, ^{1}H-NMR (400 MHz, DMSO-d$_6$) δ_H ^{1}H-NMR (400 MHz, DMSO-d6); δ_H 3.92 (3H, s), 7.38-7.44 (2H, m), 7.51-7.55 (2H, m), 7.87 (1H, t, *J*=9.6), 7.94 (1H, d, *J*=7.6), 8.22 (1H, s), 8.26 (1H, s); ^{13}C-NMR (100 MHz, DMSO-d$_6$) δ_C 55.89, 106.94, 116.48, 123.76, 124.67, 126.65, 126.76, 128.25, 128.60, 130.20, 131.06, 135.54, 148.57, 155.03, 167.59; FAB-MS *m/z* 258 [M+H]$^+$.

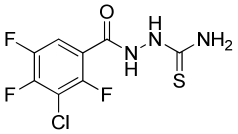

Intermediate of **32**, Amorphous powder, m.p. >200 °C, yield 57 % (method A), ^{1}H-NMR (400 MHz, DMSO-d$_6$) δ_H 7.67 (1H, s), 8.06 (2H, s), 9.53 (1H, s), 10.50 (1H, s); ^{13}C-NMR (125 MHz, DMSO-d$_6$) δ_C 111.01, 111.20, 116.84, 117.00, 118.92, 119.06, 145.02, 145.13, 147.15, 147.29, 149.19, 149.33, 161.16, 182.09; FAB-MS *m/z* 284 [M+H]$^+$.

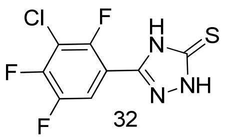

Compound **32**, Amorphous powder, m.p. >200 °C, yield 74 %, ^{1}H-NMR (400 MHz, DMSO-d$_6$) δ_H 7.95-8.03 (1H, m), 13.92 (1H, s), 13.98 (1H, s); ^{13}C-NMR (100 MHz, DMSO-d$_6$) δ_C 110.95, 111.08, 111.81, 111.98, 112.19, 115.30, 115.52, 145.30, 145.46, 146.58, 146.72, 147.75, 147.87, 149.10, 149.26, 167.46; FAB-MS *m/z* 266 [M+H]$^+$.

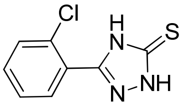

33

Known compound (**33**), 3-(2-chlorophenyl)-1H-1,2,4-triazole-5(4H)-thione synthesized by method A was agreed with published data in following article: B. Tozkoparan et al., Bioorg. Med. Chem. 15, 4, 1808-1814 (2007)

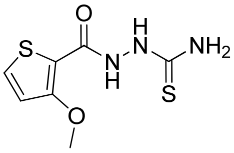

Intermediate of **34**, Amorphous powder, m.p. >200 °C, yield 41 % (method A), ^{1}H-NMR (400 MHz, DMSO-d_6) δ_H 3.96 (3H, s), 7.13 (1H, d, *J*=5.2Hz), 7.80 (1H, d, *J*=5.6Hz), 9.20 (1H, s), 9.30 (1H, s); ^{13}C-NMR (100 MHz, DMSO-d_6) δ_C 59.4, 113.7, 117.2, 131.3, 158.1, 164.7, 182.3 ; FAB-MS *m/z* 232 $[M+H]^+$.

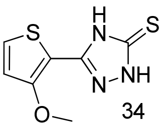

34

Compound **34**, Amorphous powder, m.p. >200 °C, yield 74 %, ^{1}H-NMR (400 MHz, DMSO-d_6) δ_H 3.90 (3H, s), 7.14 (1H, d, *J*=5.6Hz), 7.69 (1H, d, *J*=5.6Hz), ^{13}C-NMR (100 MHz, DMSO-d_6) δ_C 58.9, 103.2, 117.2, 128.4, 145.1, 157.8, 166.5; FAB-MS *m/z* 214 $[M+H]^+$.

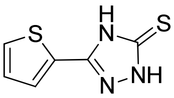

35

Known compound **35** [CAS: 68744-68-3] is commercially available products.

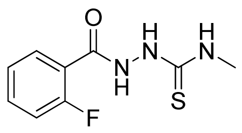

Intermediate of **36**, Amorphous powder, m.p. >200 °C, yield 62 % (method A), ^{1}H-NMR (400 MHz, DMSO-d_6) δ_H 2.90 (3H, d, *J*=4.8), 7.29 (1H, m), 7.32 (2H, m),

7.59 (1H, m), 7.83 (1H, t, *J*=6.8Hz), 7.97 (1H, s), 9.43 (1H, s), 10.13 (1H, s); ^{13}C-NMR (100 MHz, DMSO-d_6) δ_C 31.4, 116.5, 116.7, 121.9, 122.0, 124.6, 124.7, 131.0, 133.6, 133.7, 158.7, 161.2, 164.0, 182.5; FAB-MS *m/z* 228 $[M+H]^+$.

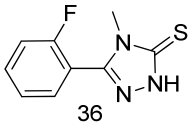

36

Compound **36**, Amorphous powder, m.p. >127-131 °C, yield 59 %, ^{1}H-NMR (400 MHz, DMSO-d_6) δ_H 3.44 (3H, s), 7.44 (1H, t, *J*=7.6), 7.50 (1H, t, *J*=10.4), 7.68 (1H, m),7.73(1H, m); ^{13}C-NMR (100 MHz, DMSO-d_6); δ_C 31.4, 114.2, 116.5, 116.7, 125.5, 132.2, 133.9, 134.0, 158.6, 161.1, 167.7; FAB-MS *m/z* 210 $[M+H]^+$.

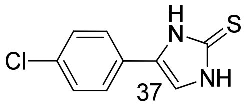

37

Known compound (**37)**, 3-(2-chlorophenyl)-1H-1,2,4-triazole-5(4H)-thione [CAS: 58755-00-3] synthesized according to following article: S. Maeda et al., Chemical & Pharmaceutical Bulletin, 32, 7, 2536-43 (1984)

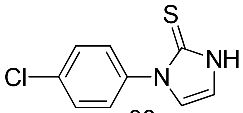

38

Known compound (**38**), 1-(4-Chlorophenyl)-1,3-dihydro-2H-imidazole-2-thione [CAS: 17452-12-9] is commercially available compound.
